# Cladistics of South American *Triatoma* (Hemiptera: Reduviidae): a morphological overview of *T. infestans* complex

**DOI:** 10.1590/0074-02760250293

**Published:** 2026-07-20

**Authors:** João Paulo Sales Oliveira-Correia, Hélcio Reinaldo Gil-Santana, Cleber Galvão

**Affiliations:** 1 Fundação Oswaldo Cruz-Fiocruz, Instituto Oswaldo Cruz, Laboratório Nacional e Internacional de Referência em Taxonomia de Triatomíneos, Rio de Janeiro, RJ, Brasil; 2 Fundação Oswaldo Cruz-Fiocruz, Instituto Oswaldo Cruz, Laboratório de Diptera, Rio de Janeiro, RJ, Brasil

**Keywords:** evolutionary relationship, subcomplexes, Triatominae, vectors, Chagas disease

## Abstract

**BACKGROUND:**

*Triatoma infestans* complex comprises 39 species of South American triatomines, organised into different subcomplexes. These species act as vectors of the protozoan *Trypanosoma cruzi*, the etiological agent of Chagas disease. However, the evolutionary relationships among the subcomplexes remain unresolved.

**OBJECTIVES:**

In this study, we present the most comprehensive morphological cladistic analysis of these species to date.

**METHODS:**

Phylogenetic relationships were examined in a new analysis based on morphological data. We analysed a data matrix comprising 48 terminal taxa and 72 morphological characters, coded under implicit parsimony weights, with a range of concavities (k1-80).

**FINDINGS:**

Our results indicate that the *T. infestans* and *Triatoma rubrovaria* subcomplexes constitute monophyletic groups, while *Triatoma brasiliensis*, *Triatoma matogrossensis* and *Triatoma sordida* subcomplexes are merophyletic. We also confirm that *Triatoma maculata* does not belong to the homonymous subcomplex.

**MAIN CONCLUSIONS:**

The study presents the first cladistic analysis and provides a solid foundation for understanding phylogenetic relationships within of the *T. infestans* complex.

Triatomines are insects of public health importance, acting as vectors of the protozoan *Trypanosoma cruzi* (Chagas, 1909),^1^ the etiological agent of Chagas disease (CD). All triatomines, commonly known as kissing bugs, belong to the subfamily Triatominae Jeannel, 1919 (Hemiptera), which has 159 species (156 living and three fossils) distributed in five tribes and 19 genera.^2^ ^3^

The genus *Triatoma* Laporte, 1832 is composed of 76 species, divided into three groups, following their geographic distributions: (i) *Triatoma rubrofasciata* group, species from Central and North America, in addition to the Old World; (ii) *Triatoma dispar* group, species from the Andes Mountains; and (iii) *Triatoma infestans* group, species from South America.^4^ ^5^ ^6^ ^7^

Species of the *T. infestans* group are classified into specific complexes based on phenotypic similarity, geographic distribution, phylogeny and epidemiology.^7^ Among these, the *T. infestans* complex encompasses a greater diversity of species, with 37 taxa, distributed into eight subcomplexes.^7^ However, these groupings have undergone modifications, and the most recent proposal indicates the following subcomplexes: *Triatoma brasiliensis*, *Triatoma costalimai*, *T. infestans*, *Triatoma maculata*, *Triatoma pseudomaculata*, *Triatoma rubrovaria*, *Triatoma sordida* and *Triatoma vitticeps*.^8^ ^9^ ^10^

Phylogenetic studies, with mitochondrial genes (with the markers 16S rDNA, 12S rDNA, cytochrome oxidase I, cytochrome oxidase II and cytochrome b), nuclear (with the markers 18S, 28S and Wingless) and ultraconserved elements, have indicated that the majority of subcomplexes do not represent a monophyletic group.^11^ ^12^ ^13^ ^14^ ^15^

However, there is still no consensus regarding the characteristics that define these species groups, and further studies are needed to corroborate or refute their validity.^9^ ^15^ ^16^ It is important to note that groups, complexes and subcomplexes are not taxonomic categories recognized by the International Code of Zoological Nomenclature; therefore, caution is recommended when proposing such groupings.^17^

In the present study, we performed a cladistic analysis to test the monophyly of the current recognised subcomplexes within *T. infestans* complex and to investigate the evolutionary relationships.

## MATERIALS AND METHODS

*Selection of terminal taxa* - The ingroup comprises all species of the *T. infestans* complex, which includes all South American species, except the five species belong to the *T. dispar* complex (Table). According to the previous hypotheses proposed by Hypša et al.,^11^ Gardim et al.,^12^ Justi et al.,^13^ ^14^ Teves et al.^18^ and Kieran et al.,^15^ and taking care to avoid the introduction of homoplasies associated with very distant taxa, the outgroup is composed by *Dipetalogaster maxima* (Uhler, 1894), *Eratyrus mucronatus* Stål, 1859, *Nesotriatoma bruneri* Usinger, 1944, *Panstrongylus megistus* (Burmeister, 1835), *Panstrongylus tibiamaculatus* (Pinto, 1926), *Panstrongylus tupynambai* Lent, 1942, *Panstrongylus rufotuberculatus* (Champion, 1899), *Psammolestes tertius* Lent & Jurberg, 1965, *Rhodnius stali* Lent, Jurberg & Galvão, 1993 and *T. rubrofasciata* (De Geer, 1773). These species are not closely related to the ingroup species and were used to polarise the characters, test the monophyly of the group, and root the resulting trees.

**TABLE:** List of material examined of the ingroup species, with respective deposit collections of types and non-type specimens

Genus	Species analysed (with status)	Depository institutions
*Triatoma*	*arthurneivai* Lent & Martins, 1940 (holotype)	CTIOC
	*bahiensis* Sherlock & Serafim, 1967 (syntype)	CTIOC
	*baratai* Carcavallo & Jurberg, 2000 (holotype)	CTIOC
	*brasiliensis* Neiva, 1911 (holotype)	CEIOC
	*carcavalloi* Jurberg, Rocha & Lent, 1998 (paratype)	CTIOC
	*circummaculata* (Stål, 1859) (syntype)*	MfN
	*costalimai* Verano & Galvão, 1958 (paratype)	CTIOC
	*deaneorum* Galvão, Souza & Lima, 1967 (holotype)	CEIB
	*delpontei* Romaña & Abalos, 1947 (paratype)	CTIOC
	*garciabesi* Carcavallo, Cichero, Martínez, Prosen & Ronderos, 1967 (holotype)	CTIOC
	*guasayana* Wygodzinsky & Abalos, 1949 (paratype)	CTIOC
	*infestans* (Klug, 1834) (non-type)	CTIOC
	*jatai* Gonçalves, Teves-Neves, Santos-Mallet, Fuente & Lopes, 2013 (paratype)	CTIOC
	*juazeirensis* Costa & Felix, 2007 (holotype)	CEIOC
	*jurbergi* Carcavallo, Galvão & Lent, 1998 (holotype)	CTIOC
	*klugi* Carcavallo, Jurberg, Lent & Galvão, 2001 (holotype)	CTIOC
	*lenti* Sherlock & Serafim, 1967 (holotype)	CTIOC
	*limai* Del Ponte, 1929 (holotype)	CTIOC
	*maculata* Erichson, 1848 (syntype)*	MfN
	*matogrossensis* (Leite & Barbosa, 1953) (non-type)	CTIOC
	*melanica* Argolo & Felix, 2006 (non-type)	CEIOC
	*melanocephala* Neiva & Pinto, 1923 (paratype)	CTIOC
	*infestans* var. *melanosoma* (Martínez, Olmedo & Carcavallo, 1987) (paratype)	CTIOC
	*oliveirai* (Neiva, Pinto & Lent, 1939) (holotype)	CTIOC
	*patagonica* Del Ponte, 1929 (non-type)	CTIOC
	*petrocchiae* Pinto & Barreto, 1925 (holotype)	CTIOC
	*pintodiasi* Jurberg, Cunha & Rocha, 2013 (holotype)	CTIOC
	*platensis* Neiva, 1913 (non-type)	CTIOC
	*pseudomaculata* Corrêa & Espínola, 1964 (holotype)	CTIOC
	*rosai* Alevi, Oliveira, Garcia, Cesaretto, Grzyb, Bittinelli, Resis, Ravazi, Bortolozo, Galvão, Azeredo-Oliveira, Tercilia & Madeira, 2020 (paratype)	CTIOC
	*rubrofasciata* (De Geer, 1773) (non-type)	CTIOC
	*rubrovaria* (Blanchard, 1843) (non-type)	CTIOC
	*sherlocki* Papa, Jurberg, Carcavallo, Cerqueira & Barata, 2002 (paratype)	CTIOC
	*sordida* (Stål, 1859) (non-type)	CTIOC
	*vandae* Carcavallho, Jurberg, Rocha, Galvão, Noireau & Lent, 2002 (holotype)	CTIOC
	*vitticeps* (Stål, 1859) (syntype)*	MfN
	*williami* Galvão, Souza & Lima, 1956 (paratype)	CEIB and CTIOC
	*wygodzinskyi* Lent, 1951 (paratype)	CTIOC

CTIOC: Coleção de Triatomíneos do Instituto Oswaldo Cruz, Rio de Janeiro, Brazil; CEIB: Coleção Entomológica do Instituto Butantan, São Paulo, Brazil; CEIOC: Coleção Entomológica do Instituto Oswaldo Cruz, Rio de Janeiro, Brazil; MfN: Hemimetabola Collection, Museum für Naturkunde, Berlin, Germany. *Analysed from photographs by Rodrigues *et al*.^48^

*Terminology, character and measurements* - The adopted terminology mainly follows Lent & Wygodzinsky,^6^ Weirauch^19^ and Schuh & Weirauch.^20^ The character survey was based on the original descriptions of each species and in publications by Galvão,^21^ Lent et al.,^22^ ^23^ ^24^ ^25^ Lent & Wygodzinsky,^6^ Carcavallo et al.,^26^ ^27^ Costa et al.,^28^ Weirauch,^19^ Obara et al.,^16^ Jurberg et al.,^29^ Gonçalves et al.,^30^ Galvão & Dale,^31^ Mendonça et al.,^32^ Rodrigues,^33^ Alevi et al.^34^ and Oliveira-Correia et al.^35^

**Fig. 1:**
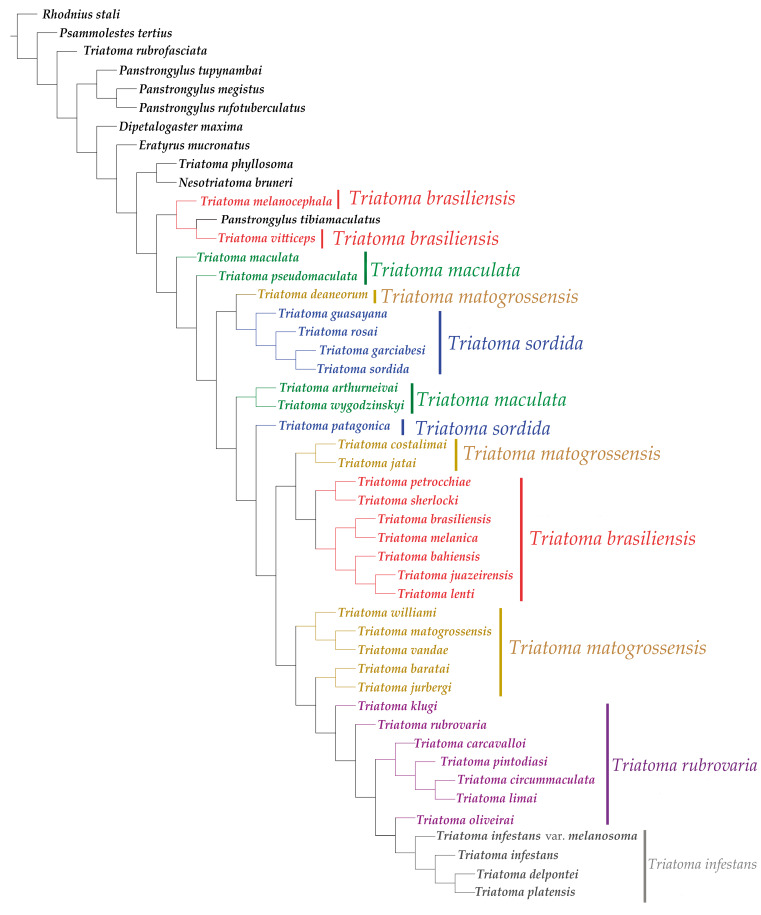
strict consensus of the 21 most parsimonious trees that resulted from the analysis under equal weights (EW) (L = 291, CI = 0.34, RI = 0.66). The classification follows.^7^ ^49^ Terminal taxa in colour: black = outgroup; red = *Triatoma brasiliensis*; green = *Triatoma maculata*; yellow = *Triatoma matogrossensis*; purple = *Triatoma rubrovaria*; grey = *Triatoma infestans*. (L = tree length, CI = consistency index, CR = retention index).

For measurements and photographs of the studied species, 213 specimens were used, of which 118 were females and 95 males [Supplementary data (Data 1)]. A Leica M205® stereoscopic microscope and a Leica DMC 2900 camera were used, with the aid of the version 4.9. All measurements are in millimetres (mm) [Supplementary data (Data 2, Fig. 1A-C)]. For the rostrum sensu Lent and Wygodzinsky,^6^ we will use the term labium, as it has been used in more recent literature.^20^ In almost all subfamilies of Reduviidae, including Triatominae, the first labial segment is lost or fused to the head capsule and is therefore not externally visible; the three visible segments correspond to segments II, III and IV.^29^

The characters were coded by contingency,^36^ following the standards of Sereno^37^ and Brazeau.^38^ The following characters have been encoded. The character matrix was created using the Mesquite 3.81 program^39^ [Supplementary data (Table I)].

*Phylogenetic analysis* - We submitted the data set to a parsimony analysis under equal weights (EW) and implied weights (IW) in TNT 1.6.^40^ The IW analysis carried the searches out under RAS+TBR, with a wide range of concavities (k3 to k80). The approach used here derives from Goloboff et al.,^41^ being the basic strategy to explore different parameters of the concavity constant (k) and group the searches when the Most-Parsimonious Trees (MPTs) are found, to investigate the effect of character weighting on hypotheses of relationships.

To make a robust and exhaustive analysis, the parameters used in all searches were 5000 TBR replications with 1000 trees saved per replication. To calculate the nodal support, we used TNT through bootstrap Poisson independent re-weighting and relative Bremer support.^42^

## RESULTS

**Fig. 2:**
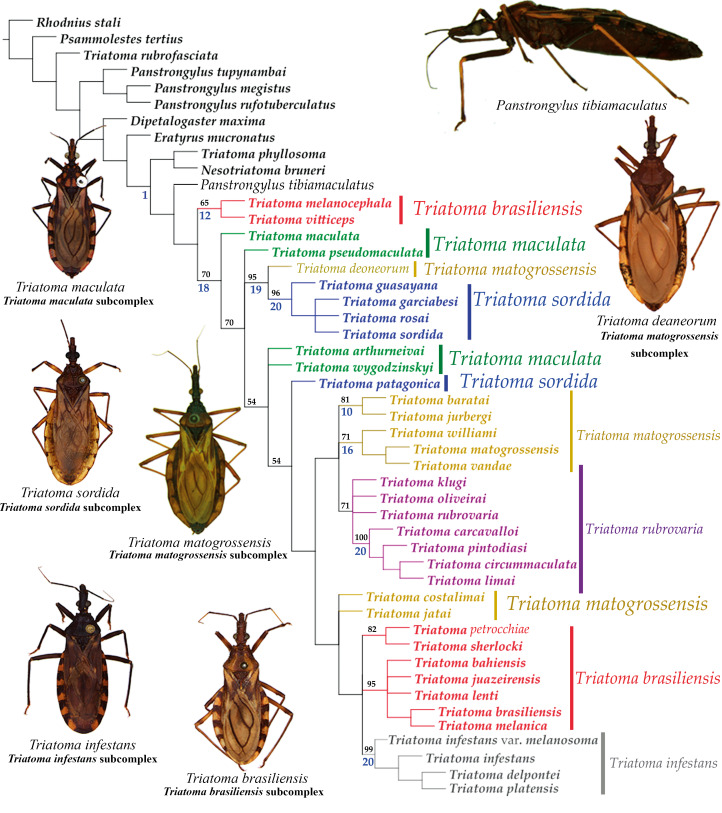
strict consensus of the three most parsimonious trees that resulted from the analysis under implied weights (IW) with K = 3 (Fit = 2.18956, L = 250, CI = 0.41, RI = 0.59). Bremer support values (> 50) mapped above branches and bootstrap values over 50% are given below the nodes. Blue numbers on nodes (1-21) represent clades. The classification follows.^7^ ^49^ Terminal taxa in colour: black = outgroup; red = *Triatoma brasiliensis*; green= *Triatoma maculata*; yellow = *Triatoma matogrossensis*; purple = *Triatoma rubrovaria*; grey = *Triatoma infestans*. (L = tree length, CI = consistency index, CR = retention index).

The data matrix comprised 48 terminal taxa and 72 characters were encoded: 49 binary and 23 multistate [Supplementary data (Table II)]. The analysis recovered all Triatomini species studied, with 13 supported synapomorphies for both the EW and IW clades [Supplementary data (Fig. 2)]. EW analysis result in 21 MPTs (with L = 291, CI = 0.34, RI = 0.66) (Fig. 1).

For the IW analysis, we observed 12 strict consensus MPTs with the same topology. We chose the tree under k3 (Fig. 2, Fit = 2.18956, L = 250, CI = 0.41, RI = 0.59) for graphical representation, to map the *Bootstrap* and *Bremer* support values, to obtain character scores and basis for discussion. The disagreement between the EW and IW analyses was limited to the position of *Triatoma oliveirai* and the presence of polytomy in clades of the *T. sordida*, *T. rubrovaria* and *T. brasiliensis* subcomplexes (Fig. 2).

The *Triatoma* species recovered as a monophyletic group, except for *Triatoma phyllosoma* and *T. rubrofasciata*. However, *P. tibiamaculatus* is grouped close to the clade (*T. vitticeps* + *Triatoma melanocephala*) and distant from the other *Panstrongylus* species Berg, 1879 (Fig. 2).

**Fig. 3:**
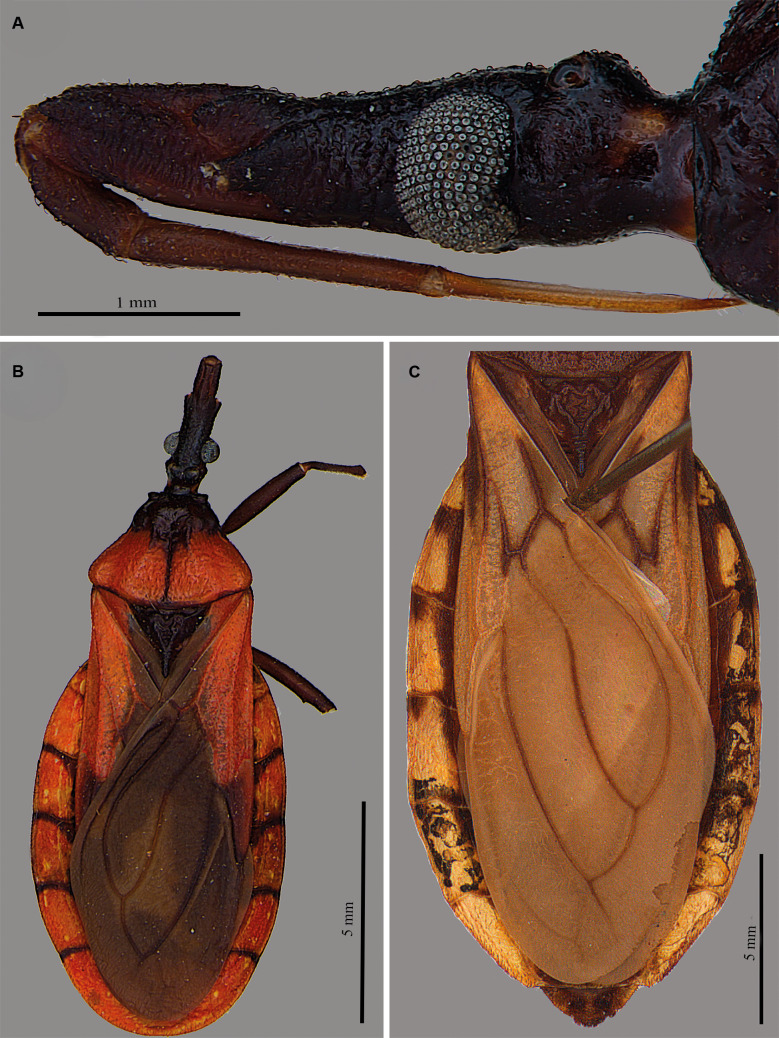
characters highlighted in the analysis. (A) *Triatoma pintodiasi*, head, lateral view. (B) *T. pintodiasi*, dorsal view. (C) *Triatoma deaneorum*, posterior portion of the body, including its connexivum, dorsal view.

We observed that the *T. rubrovaria* and *T. infestans* subcomplexes form monophyletic groups (Fig. 2). For the *T. rubrovaria* subcomplex, the clade (*Triatoma pintodiasi* + (*Triatoma circummaculata* + *Triatoma limai*) was supported by a synapomorphy: 32 (1), third visible segment of the labium as long as or longer than the second. It is worth noting that *T. pintodiasi* has the apomorphy (Fig. 3A); 9 (1), pronotum with predominantly orange or yellowish posterior lobe (Fig. 3B) [Supplementary data (Fig. 3.10)].

Therefore, the *T. brasiliensis* subcomplex is not a monophyletic group, because *T. vitticeps* and *T. melanocephala* are distant from the other species of the subcomplex. Similarly, the *T. maculata* subcomplex is polyphyletic, with (*Triatoma arthurneivai* + *Triatoma wygodzinskyi*) being distant from *T. maculata* and *T. pseudomaculata* (Fig. 2).

The *Triatoma matogrossensis* and *T. sordida* subcomplexes are also not monophyletic groups (Fig. 2). *Triatoma deaneorum* is grouped in the same clade as most species of the *T. sordida* subcomplex, supported by a synapomorphy: 11 (1), pronotum, connexivum, in dorsal view, with dark spots in the shape of musical notes on the intersegmental suture (Fig. 3C). This clade is formed by all species of the *T. sordida* subcomplex, except *Triatoma patagonica*. On the other hand, the species of the *T. matogrossensis* subcomplex are close (*Triatoma baratai* + *Triatoma jurbergi*) and (*Triatoma williami* + (*T. matogrossensis* + *Triatoma vandae*), except *T. costalimai* and *Triatoma jatai*.

## DISCUSSION

Among the characters used in the analysis, as well as previous taxonomic studies of triatomines, the connexivum spot pattern is a character frequently used in the comparison or diagnosis of species. However, recent studies have shown phenotypic variations in this character, which has resulted in the synonymies between some taxa (*e.g.*, *Triatoma melanosoma* Lent, Jurberg, Galvão & Carcavallo, 1994 = *T. infestans*, currently *T. infestans* var. *melanosoma*)^24^ and (*Triatoma guazu* Lent & Wygodzinsky, 1979 = *T. williami*).^35^ In the present analysis, this character was used with caution and always in agreement with that presented in the type specimens examined, especially the primary types (name-bearing).

Our study presents the first evolutionary hypothesis based on morphological characters that encompasses most South American *Triatoma* species. The phylogenetic reconstructions included a larger number of taxa, including rare species that had been overlooked in previous studies. However, previous molecular analyses failed to clarify the deeper evolutionary relationships among clades. According to Justi et al.,^13^ state relationships within subcomplexes remain unresolved.

According to Schofield & Galvão,^7^ *T. melanocephala*, *Triatoma tibiamaculata* (currently, *P. tibiamaculatus* nov. comb.) and *T. vitticeps* make up the *T. brasiliensis* subcomplex. Previous hypotheses have observed the close relationship between *T. melanocephala* and *T. vitticeps*.^15^ However, Alevi et al.,^9^ based on morphological, phenotypic and genotype characteristics (cytogenetic and molecular), proposed the establishment of the *T. vitticeps* subcomplex (*T. melanocephala*, *P. tibiamaculatus* and *T. vitticeps*), since these species were distinct from all other subcomplexes of the *T. infestans* complex.

Phylogenetic analyses, based on molecular and cytogenetic data, have revealed a highly supported close relationship of *P. tibiamaculatus* with *P. megistus*.^13^ ^15^ ^43^ According to Rodrigues,^33^ these two species present the same characteristics in the terminal abdominal segments of the females (abdominal segments IX and X combined in the form of an elongated trapezoid) and (abdominal segments VII-X combined longer than wide), differentiating it from other South American *Triatoma* species. Our topology did not recover *P. tibiamaculatus* and *P. megistus* as sister species; however, we consider the morphological characteristics shared between *P. tibiamaculatus* and *Triatoma* spp. to represent homoplasies as suggested by Bitinelli et al.^43^

The *T. brasiliensis* subcomplex was redefined as a monophyletic group composed of the species: *T. brasiliensis*, *Triatoma juazeirensis*, *Triatoma melanica*, *Triatoma lenti*, *Triatoma petrocchiae* and *Triatoma sherlocki*.^44^ Here *T. petrocchiae* stands out as a sister species to the other species of the *T. brasiliensis* subcomplex, corroborating the hypotheses of Oliveira et al.^44^ According to Kieran et al.,^15^ *T. petrocchiae* appears as a sister species to the clade [*T. baratai* + (*T. williami* + *T. guazu*) + (subcomplexo *T. sordida*)], as observed in the trees generated by data from Ultraconserved Elements + Ribosomal and morphological (based on chromatic pattern and presence of fossula spongiosa). In our morphological analysis, we verified the absence of fossula spongiosa in *T. petrocchiae* and *T. sherlocki*, which corroborates the hypothesis proposed by Kieran et al.^15^ It is worth noting that the absence of fossula spongiosa is an ancestral characteristic, and their presence is a characteristic present in most Triatominae lineages.

There is no consensus regarding the *T. maculata* subcomplex, *T. maculata* consistently appears more distant from the other species within the subcomplex.^13^ ^15^ According to Galvão et al.,^21^ *T. maculata* and *T. pseudomaculata* can be distinguished by total body length, spot pattern and male genitalia. In our analysis, we highlighted the following characters for both species: presence of spots on the head; width of the eye in relation to the dorsal interocular space; size of the anterolateral angles; and presence of basal disc tubercles on the anterior lobe of the pronotum. According to Justi et al.,^14^ the separation of *T. maculata* from the other *Triatoma* species in the Southern Hemisphere, except for *T. melanocephala* and *T. vitticeps*, in agreement with our analyses, is associated with the uplift of the Northern Andes (23-10 Ma). Consequently, the ancestral population of *T. maculata* was restricted to the Guiana Shield.

*Triatoma arthurneivai*, *T. pseudomaculata* and *T. wygodzinskyi* are grouped in the *T*. *maculata* subcomplex.^7^ Our morphological analysis recovered these three species at distant terminals, except for the clade (*T. arthurneivai* + *T. wygodzinskyi*). However, according to Lent & Wygodzinsky,^6^ *T. arthurneivai* and *T. wygodzinskyi* are very similar morphologically, but can be distinguished by the male genitalia, spot pattern of the pronotum and corium of the hemelytron. Such characters were not used in the current analysis, as they presented many intraspecific variations. Thus, as suggested by Masarin et al.,^10^ the existence of the new *T. pseudomaculata* subcomplex was not corroborated in our morphological analysis.

Previous studies indicate that the *T. matogrossensis* subcomplex should be disregarded, as it represents a paraphyletic group, with its species relocated to the *T. sordida* and *T. maculata* subcomplexes.^8^ ^12^ ^13^ ^15^ Likewise, existing hypotheses concerning the *T. sordida* subcomplex suggest a close relationship with some of the species of the *T. matogrossensis* subcomplex (*i*. *e.*, *T. matogrossensis* e *T. vandae*),^8^ ^12^ ^13^ ^15^ ^18^ which was not supported by our analysis. We consider our study to provide a more comprehensive perspective, as it includes morphological data from all species of the *T. sordida* subcomplex and *T. matogrossensis*, encompassing rare species and those not previously included in phylogenetic analyses.

In the present analysis *T. deaneorum*, little-reported species not analysed in existing hypotheses, was included.^12^ ^13^ ^14^ ^15^ *T. deaneorum* proved to be a sister taxon of [*Triatoma guasayana* + (*Triatoma rosai* + *Triatoma garciabesi* + *T. sordida*)], forming a clade supported by one synapomorphy: 11(1), spot pattern of the dorsal connexivum plates in the form of musical notes, covering the intersegmental sutures. In contrast, Lent and Wygodzinsky^6^ suggested this species was a hybrid of *T. infestans* and *T. williami*. However, our phylogenetic topologies do not refute a close relationship between this species and *T. deaneorum*.

The close relationship of *T. garciabesi*, *T. rosai* and *T. sordida* was the same for EW and IW. About two decades, *T. garciabesi* remained synonymous with *T. sordida*.^6^ These two species were separated by morphological, morphometric, isoenzymatic, cytogenetic and phylogenetic studies, resulting in the revalidation of *T. garciabesi*.^45^ ^46^ ^47^

*Triatoma rosai* was described by an integrative approach, using morphological, morphometric, molecular data and experimental crossings.^34^ Our study confirms the delimitation of *T. rosai* (previously termed as intraspecific variation of *T. sordida* from Argentina) and *T. sordida sensu stricto*.^34^ The following diagnostic character was highlighted: for *T. sordida*, the posterior process of the scutellum is as long as or longer than the main body of the scutellum, whereas in *T. rosai* it is shorter.

*Triatoma costalimai* and *T. jatai* are considered part of the *T. matogrossensis* subcomplex.^5^ According to Pita et al.,^8^ these two species would be grouped into the new *T. arthurneivai* subcomplex (current *T. pseudomaculata* subcomplex proposed by Masarin et al.^10^), which does not corroborate our hypothesis. However, Masarin et al.,^10^ based on experimental crosses and molecular data, proposed the creation of the new *T. costalimai* subcomplex composed of *T. costalimai* and *T. jatai*. These two species are morphologically similar and can be differentiated by the proportion and general coloration of the body, the pattern of connective tissue spots, hairiness on the labial segments, and the shape of the male genitalia.^30^ In the present analysis, *T. costalimai* and *T. jatai* were recovered as sister species and highlighted a new diagnostic character for such species: for *T. jatai*, submedian carina of the pronotum, reaching the distal margin of the posterior lobe and, for *T. costalimai*, limited to the median region of the posterior lobe.

Currently, there is no consensus on which and how many species are part of the *T. rubrovaria* subcomplex.^8^ ^13^ ^14^ ^15^ According to Pita et al.^8^ and Kieran et al.,^18^ *T. patagonica* and *T. guasayana* make up the *T. rubrovaria* subcomplex, which was not observed in the current analysis and previous studies.^8^ ^12^ ^15^ ^47^

Among the species of the *T. rubrovaria* subcomplex it was possible to observe the clade (*T. pintodiasi* + (*T. circummaculata* + *T. limai*)). According to Jurberg et al.,^29^ these three species present marked morphological similarity, since our analyses indicate the following diagnostic characters for *T. circummaculata* and *T. limai*: predominant coloration of the posterior lobe, pattern of spots on the dorsal plates of the connexivum, length of the third antennal segment in relation to the second and the shape of the posterior process of the scutellum.

All species of the *T. infestans* subcomplex were established in both EW and IW analyses. The EW reconstruction showed *T. oliveirai* in the same clade as the other species of the *T. infestans* subcomplex. Although additional evidence is required, *T. oliveirai* may belong to this a subcomplex, as the species occur sympatrically in southern Brazil. We can observe that the *T. infestans* subcomplex was monophyletic in all analyses, but we cannot clearly highlight the evolutionary relationship with the other species studied.

However, we highlight that: (i) the *T. infestans* and *T. rubrovaria* subcomplexes form monophyletic groups; (ii) the *T. brasiliensis* subcomplex proved to be polyphyletic: *T. melanocephala* and *T. vitticeps* form a distant clade and *T. sherlocki* and *T. petrocchiae* form a clade sister to most of the species in the subcomplex; (iii) we confirmed the inappropriateness of the *T. matogrossensis* subcomplex and the close relationship of *T. deaneorum* with the *T. sordida* subcomplex; (iv) *T. maculata* is not part of the subcomplex that bears its name; and (v) *T. patagonica* is not included in the *T. sordida* subcomplex.

In conclusion, this study presents the first comprehensive cladistic analysis of the *T. infestans* complex, including all species, except for the recently described *Triatoma chiarii*.^3^ This work provides a solid foundation for understanding the phylogenetic relationships within this complex.

## SUPPLEMENTARY MATERIALS

Supplementary material

## Data Availability

The present study examined adult specimens of both sexes [Supplementary data]. All specimens are deposited in the following entomological collections: CTIOC - Coleção de Triatomíneos, Instituto Oswaldo Cruz (Fiocruz), Rio de Janeiro, Brazil; CEIOC - Coleção Entomológica, Instituto Oswaldo Cruz (Fiocruz), Rio de Janeiro, Brazil; CEIB - Coleção Entomológica, Instituto Butantan, São Paulo, Brazil; MfN - Hemimetabola Collection, Museum für Naturkunde, Berlin, Germany.
