# Supplementary material for: Cladistics of South American Triatoma (Hemiptera: Reduviidae): a morphological overview of T. infestans complex
Source: Mem Inst Oswaldo Cruz. 2026 Jul 20;121(Suppl 1):e250293. doi: 10.1590/0074-02760250293 (PMC13395083; doi:10.1590/0074-02760250293)
Supplement: Supplementary material [file 1678-8060-mioc-121-s1-e250293-s1.pdf]

Data 1. Examined material of the studied species

A forward slash (/) separates lines, a double forward slash (//) identifies different labels, and a semicolon (;) separates specimens. The material is listed according to the original sample label.

Ingroup
Triatoma arthurneivai Lent & Martins, 1940

Holotype
Triatoma arthurneivai/ Lent e Martins, 1940/ n. sp. Holótipo ♀/ 8.940 H. LENT DET.// Serra do Cipó/ Faz. Alto do Pala/ cio - Minas Gerais/ Aurilecar coll. 3/940// sobre muro/ de pedras/ soltas// Holotypus// Holótipo// N. 1053/ HEMIPTERA/ Inst. Oswaldo Cruz// CTIOC/ N° 13147

Paratypes
Triatoma arthur/ neivai Lent &/ Martins/ H. LENT det.// Serra do Cipó/ Minas/ a. Martins// ALLO/ TYPUS// N° 1054/ HEMIPERA/ Inst. Oswaldo Cruz// Parátipo// CTIOC N° 8952; *Triatoma arthur/ neivai* Lent &/ Martins/ H. LENT det.// Serra do Cipó/ Minas/ a. Martins// detalhe// N° 1055/ HEMIPERA/ Inst. Oswaldo Cruz// Parátipo// CTIOC N° 9909

Triatoma bahiensis Sherlock & Serafim, 1967

Syntype
Triatoma/ bahiensis/ det. I. Sherlock III. 1967// *Triatoma lenti*/ Sherlock e Serafim/ 8.975 H. LENT det.// Seabra/ Bahia/ 1950// Paratypus// Sintipo// Parátipo// N° 2774// CTIOC/ N° 3338

Triatoma baratai Carcavallo & Jurberg, 2000

Holotype
FIOCRUZ/ Instituto Oswaldo Cruz/ T. baratai/ sp. n./ Ex 5631/ Col. Carcavallo// BRASIL - Mato Grosso/ Sul/ Local Mun Bonito/ Col. E. B. Galati/ Data 30.7.98/ I.O.Cruz// HOLOTIPO// Holótipo// CTIOC/ N° 9796

Other material
T. baratai/ C. Galvão/ det. III-2004// *Triatoma williami*/ 7-79 Miranda/ Mato Grosso/ H. LET det./ domic.// Coleção Herman Lent/ Laboratório Nacional e/ Internacional de Referência em/ Taxonomia de Triatomíneos// CTIOC/ N° 11087; *Triatoma baratai*/ Det. Ports, 2008/ 1747// BRASIL -Mato Grosso do Sul/ Bonito, Faz. Bodoquema/ Galinheiro no peridomicílio/ Col. Rosa D. da 8.xi.2007// ♂// Coleção Herman Lent/ Laboratório Nacional e/ Internacional de Referência em/ Taxonomia de Triatomíneos// CTIOC/ N° 1210; *Triatoma/ baratai*/ J. Jurberg/ 3. 2010// BRASIL – MS/ Corumba/ LATEC/ 8.2006// criado no/ insetário/ LNIRTT/ 3-2010// Coleção Herman Lent/ Laboratório Nacional e/ Internacional de Referência em/ Taxonomia de Triatomíneos// CTIOC/ N° 13149; *Triatoma baratai*/ Det. Ports, 2008/ 1748// BRASIL -Mato Grosso do Sul/ Bonito, Faz. Bodoquema/ Galinheiro no peridomicílio/ Col. Rosa D. da 8.xi.2007// ♂// Coleção Herman Lent/ Laboratório Nacional e/ Internacional de Referência em/ Taxonomia de Triatomíneos// CTIOC/ N° 1211

Triatoma brasiliensis Neiva, 1911

Holotype
Instituto Oswaldo Cruz/ N 2.929/ Supporte 51/ ♀ Tipo de/ *Triatoma/ brasiliensis*/ Neiva, 1911/ Do Rio G. do/ Norte, Brasil // -Tipo-/ R. G. Norte/ Brasil

Other material
T. brasiliensis/ Neiva, 1911/ Det. J. Jurberg/ out.77// BRASIL-ESTADO PIAUI/ Local Brejo Seco/ Mun. São João Piaui/ F. Pires/ out.77// Coleção Herman Lent/ Laboratório Nacional e/ Internacional de Referência em/ Taxonomia de Triatomíneos // 2890 // CTIOC N° 6063; *T. brasiliensis*/ Neiva, 1911/ J. Jurberg/ out/ 77// BRASIL-ESTADO PIAUI/ Local Brejo Seco/ Mun. São João Piaui/ F. Pires/ out.77// Coleção Herman Lent/ Laboratório Nacional e/ Internacional de Referência em/ Taxonomia de Triatomíneos// CTIOC/ N° 6110

Triatoma carcavalloi Jurberg, Rocha & Lent, 1998

Paratype
Triatoma/ carcavalloi/ sp.n. 1998/ J. Jurberg det// BRASIL – ESTADO RS/ Local Mun. Cangaçu, Florida Povoado 27/ Col criado em lab/ R. Carcavallo// PARATPO/ 5506// Parátipo// CTIOC/ N° 9497; *Triatoma/ carcavalloi*/ sp.n. 1998/ J. Jurberg det// BRASIL – ESTADO RS/ Local Mun. Jaguarão, Corraldão Pedrosa/ Fazenda/ Col criado em lab./ R. Carcavallo// PARATPO/ 5507// Parátipo// CTIOC/ N° 9498; *Triatoma carcavalloi*/ Jurberg, Rocha & Lent, 1998/ Det// BRASIL/ Pov. Fernão Dias/ São Gerônimo-RS/ 01.xii.03// Coleção Herman Lent/ Laboratório Nacional e/ Internacional de Referência em/ Taxonomia de Triatomíneos// Reared in laboratory/ by LNIRTT/ Rio de Janeiro, RJ/ Inst. OswaldoCruz// CTIOC/ N° 14670

Triatoma circummaculata (Stål, 1859)

Syntype
circum-/ maculatus/ Stål// Buen. Ayr. Bes// 2935 [Hemimetabola Collection, Museum für Naturkunde, Berlin, Germany]

Other material
Coleção Herman Lent/ Laboratório Nacional e/ Internacional de Referência em/ Taxonomia de Triatomíneos// LACEN.RS/ Sítio Pareina/ Piratini-RS// (070645)/ 122// CTIOC/ N° 14593; *Triatoma/ circummaculata* (Stål, 1859)// Coleção Herman Lent/ Laboratório Nacional e/ Internacional de Referência em/ Taxonomia de Triatomíneos// (020077)/ 126// CTIOC/ N° 14597; *Triatoma/ circummaculata*/ (Stål, 1859)// Coleção Herman Lent/ Laboratório Nacional e/ Internacional de Referência em/ Taxonomia de Triatomíneos// CTIOC/ N° 14602

Triatoma costalimai Verano & Galvão, 1958

Paratypes
Triatoma costa-/ limai Galvão/ H. LENT det.// Parátipo// CTIOC/ Nº 9941; *Triatoma costa-/ limai* Galvão/ H. LENT det.// Mambaí/ Goiás/ Schofield col./ 7-947// Parátipo// 2720// CTIOC/ Nº 9942; *T. costalimai*/ S. Desidonio e Bahia/ det. I. Sherlock ???. 1975// Parátipo// Paratipo// CTIOC/ N° 9847

Triatoma deaneorum Galvão, Souza & Lima, 1967

Holotype
Triatoma deanei// Mun. de Piranhas, Estado de Goiás, Brasil, Coleção Entomológica do Instituto Butantan// N° 2024

Other material
Triatoma deaneorum// Mun. Chapada dos Guimarães, Mato Grosso, Brasil// Coleção Rodolfo Carcavallo// 5634

Triatoma delpontei Romaña & Abalos, 1947

Paratypes
Triatoma delpontei/ Romaña & Abalos/ 1947/ H. Lent DET.// T. platensis ♂/ IMR 344/ El Majon/ (Stago. del E.)/ 17-3-45// N. 1169/ HEMIPTERA/ Inst. Oswaldo Cruz// PARÁTIPO// Parátipo// CTIOC N° 9940; *Triatoma delpontei*/ Romaña & Abalos/ 1947/ H. Lent DET.// *T. platensis* ♀/ IMR 344/ El Majon/ (Stago. del E.)/ 17-3-45// N. 1170/ HEMIPTERA/ Inst. Oswaldo Cruz// PARÁTIPO// Parátipo// CTIOC N° 9941

Triatoma garciabesi Carcavallo, Martínez & Peláez, 1967

Holotype
Triatoma/ garciabesi/ sp nov/ R. Carcavallo det 1965// J. Cicero/ J. Hernandez/ R. Carcavallo Col.// Argentina/ Corcoba/ B. Chancani/ XII-64// FIOCRUZ/ Instituto Oswaldo Cruz/ Coleção/ Rodolfo/ Carcavallo// HOLOTYPUS/ CTIOC/ N° 13187

Paratypes
Triatoma/ garciabesi/ sp nov./ R. Carcavallo det 1965// Argentina/ Rioja/ Mascabia/ VIII-62// Coleção/ Rodolfo Carcavallo// Parátipo// Paratipo// 5243// CTIOC/ Nº 9720; *Triatoma/ garciabesi*/ sp nov./ R. Carcavallo det 1965// Argentina/ S. del Esterno/ Sancho Corral/ VIII-62// Coleção/ Rodolfo Carcavallo// Parátipo// Paratipo// 5244// CTIOC/ Nº 9721

Triatoma guasayana Wygodzinsky & Abalos, 1949

Paratypes
T. guasayana/ Det. F. Noireau/ Data VII-97/ N°// FIOCRUZ/ Instituto Oswaldo Cruz/ Tita/ Way to Izozog (Chaco)/ Bolivia 10.96/ F. Noireau// Captured/ by light. Trap// Cdca 103// Parátipo// CTIOC/ N° 9849; *Triatoma/ guasayana*/ Wygodzinsky & Abalos/ Wygodzinsky det./ Inst. MED. REG./ Paratipo// Parátipo// Parátipo// 0764/ E. calias// 0160// 2620// CTIOC/ N° 9806; *Triatoma/ guasayana*/ Wygodzinsky & Abalos/ Wygodzinsky det./ Inst. MED. REG./ Paratipo// Parátipo// Parátipo// 0152// 2623// CTIOC/ N° 9809; *Triatoma/ guasayana*/ Wygodzinsky & Abalos/ Wygodzinsky det./ Inst. MED. REG./ Paratipo// Parátipo// Parátipo// 0159// 2624// CTIOC/ N° 9810; *Triatoma/ guasayana*/ Wygodzinsky & Abalos/ Wygodzinsky det./ Inst. MED. REG./ Paratipo// Parátipo// 50602/ z T. sordida/ S. MAZZA-Coll. Det. leg// 2625// CTIOC/ N° 9811

Other material
T. guasayana/ N° FIOCRUZ 2718

Triatoma infestans (Klug, 1834)

Other material
Triatoma/ infestans ♀/ Det. J. Jurberg/ Data III. 1977/ N° FIOCRUZ// Encruzilhada/ do Sul/ R. S. – Brasil/ III.77// CTIOC/ N° 9856; *T. infestans*/ Det. F. Moirean leg./ Data 10.91/ N° FIOCRUZ// Tita/ Way to Izozog (Chaco)/ Bolívia 10.96/ F. Noireau// Silvatic from/ jarrot-nest// J1// CTIOC/ N° 9854

Triatoma infestans var. *melanosoma* Martinez, Olmedo & Carcavallo, 1987

Allotypes
Triatoma infestans/ melanosoma/ ssp. nov./ R. Carcavallo det. 1987// Allotypus// Argentina/ Misiones/ Sta. Inácio/ Col. Sta Rosa/ III-84// FIOCRUZ/ Instituto Oswaldo Cruz// Coleção/ Rodolfo/ Carcavallo// 4073// Parátipo// CTIOC/ N° 9737; *Triatoma/ melanosoma*/ ssp. nov./ R. Carcavallo det. 1987// Allotypus// Argentina/ Misiones/ Sta. Inácio/ Col. Sta Rosa/ III-84// FIOCRUZ/ Instituto Oswaldo Cruz// Coleção/ Rodolfo/ Carcavallo// 4074// Parátipo// CTIOC/ N° 9738; *Triatoma/ melanosoma*/ ssp. nov./ R. Carcavallo det. 1987// Allotypus// Argentina/ Misiones/ Sta. Inácio/ Col. Sta Rosa/ III-84FIOCRUZ/ Instituto Oswaldo Cruz// Coleção/ Rodolfo/ Carcavallo// 4076// Parátipo// CTIOC/ N° 9740

Triatoma jatai Gonçalves, Teves-Neves, Santos-Mallet, Carbajal-de-la-Fuente & Lopes, 2013

Paratypes
Triatoma jatai ♀/ Estado Tocantis/ Paranã/ Local Assentamento Jatai/ Ecot afloramento rochoso/ Col. Mallet, J. S. Data XI-2005// Parátipo// Coleção Heman Lent/ Nº 3402// CTIOC/ N° 13192; Espécie *Triatoma jatai* ♀/ Estado Tocantis/ Paranã/ Local Assentamento Jatai/ Ecot afloramento rochoso/ Col. Lopes, E. M. Data VI-2005// Parátipo// Parátipo// Coleção Heman Lent/ Nº 34030// CTIOC/ N° 13193; Espécie *Triatoma jatai* ♀/ Estado Tocantis/ Paranã/ Local Assentamento Jatai/ Ecot afloramento rochoso/ Col. Mallet, J. S. Data VIII-2005// Parátipo// Coleção Heman Lent/ Nº 3404// CTIOC/ N° 13194

Triatoma juazeirensis Costa & Félix, 2007

Holotype
Triatoma juazeirensis/ Costa & Felix, 2007/ Mun. Juazeiro/ Bahia/ Brasil/ (9°24’42”S, 40°29’55”)

Other material
Triatoma/ juazeirensis/ Costa & Felix, 2007/ ♀// Brasil: Bahia/ Itaguaçu/ Em toca do/ Cisne 24.vii.2009// CTIOC N° 3568; *Triatoma/ juazeirensis*/ Lima-Neiva, V. det.// 19-07-2011// 18-06-1996/ Almeida CE col.// CEIOC 1340

Triatoma jurbergi Carcavallo, Galvão & Lent, 1998

Holotype
Triatoma/ jurbergi/ sp n/ R. Carcavallo det. 1987// BRASIL – ESTADO/ Local Rondonopo/ lis/ Col. FNS/ Data IX-97/ N° 2903// Holotype// Holótipo// 2903// CTIOC/ N° 9815

Paratypes
Triatoma/ jurbergi/ sp n/ R. Carcavallo det. 1987// BRASIL – ESTADO/ Local Rondonopo/ lis/ Col. FNS/ Data IX-97/ N° 2904// Alotype// Parátipo// 2903// CTIOC/ N° 9816; *Triatoma/ jurbergi*/ sp n/ R. Carcavallo det. 1987// BRASIL – ESTADO/ Local Rondonopo/ lis/ Col. FNS/ Data IX-97/ N° 2906// Paratype// Parátipo// 2905// CTIOC/ N° 9817; *Triatoma/ jurbergi*/ sp n/ R. Carcavallo det. 1987// BRASIL – ESTADO/ Local Rondonopo/ lis/ Col. FNS/ Data IX-97/ N° 2906// Paratype// Parátipo// 2903// CTIOC/ N° 9818; *Triatoma/ jurbergi*/ sp n/ R. Carcavallo det. 1987// BRASIL – ESTADO/ Local Rondonopo/ lis/ Col. FNS/ Data IX-97/ N° 2906// Paratype// Parátipo// 2907// CTIOC/ N° 9819

Triatoma klugi Carcavallo, Jurberg, Lent & Galvão, 2001

Paratypes
FIOCRUZ/ Instituto Oswaldo Cruz/ Triatoma klugi/ N° 2929// BRASIL-RS/ Local: Nova Petropólis/ C. I.C Pinto e/ Col. M. Steindel/ Data 29.I.1999/ I.O.Cruz// Parátipo// Parátipo// CTIOC/ N° 9456; FIOCRUZ/ Instituto Oswaldo Cruz/ *Triatoma klugi*/ N° 2930// BRASIL-RS/ Local: Nova Petropólis/ C. I.C Pinto e/ Col. M. Steindel/ Data 29.I.1999/ I.O.Cruz// Parátipo// Parátipo// CTIOC/ N° 9457; FIOCRUZ/ Instituto Oswaldo Cruz/ *Triatoma klugi*/ N° 2931// BRASIL-RS/ Local: Nova Petropólis/ C. I.C Pinto e/ Col. M. Steindel/ Data 29.I.1999/ I.O.Cruz// Parátipo// Parátipo// CTIOC/ N° 9458;

Triatoma lenti Sherlock & Serafim, 1967

Holotype
Triatoma/ lenti/ det. Sherlocki IV 1967// Macaubas- Ba/ Col. N. P. B IV.66// *Triatoma lenti*/ Gonçalves et al. 1993// TYPUS// Holótipo// N 2770/ HEMIPTERA/ Inst. Oswaldo Cruz// CTIOC/ N° 3334

Paratypes
Triatoma lenti/ Sherlock &/ Serafim, 1967// Ipupiara/ Bahia/ 1966// *Triatoma/ pessoai*/ det. I. Sherlock III. 1967// 16// Síntipo// Paratypus// Parátipo// N. 2775/ HEMIPTERA/ Ins. Oswaldo Cruz// CTIOC/ Nº 3329; *Triatoma lenti*/ Sherlock &/ Serafim, 1967// Ipupiara/ Bahia/ 1966/ 15// *Triatoma/ pessoai*/ det. I. Sherlock III. 1967// 16// Síntipo// Paratypus// Parátipo// N. 2777/ HEMIPTERA/ Ins. Oswaldo Cruz// CTIOC/ Nº 3331

Triatoma limai Del Ponte, 1929

Holotype
T. limai n. sp.// tipo Del Ponte 1929// Brasil// Typus// Holótipo// N. 2763/ HEMIPTERA/ Inst. Oswaldo Cruz// CTIOC/ N° 13196

Triatoma maculata (Erichson, 1848)

Syntype
maculatus Er./ Stoll. F. 140/ Brit. Guy. Schomb.// Type// 2922 [analysed by photographs, Hemimetabola Collection, Museum für Naturkunde, Berlin, Germany]

Other material
Triatoma macu-/ lata (Erickson)/ H. LENT. DET.// Curacao/Piscadera baai/ IX-58-II-1959/ leg. J. H. Stook// 4// CTIOC/ Nº 4295; Surumú/ Terr. Rio Branco/ Inst. Nac. Pesq./ Amazonica 1956// 2// CTIOC/ N° 4292/; Venezuela/ San Juan de/ los Marcos/ 3-953/ Tonealba leg.// 3// CTIOC/ N° 4293

Triatoma matogrossensis Leite & Barbosa, 1953

Other material
Triatoma/ matogrossensis/ Leite & Barbosa, 1953// BRASIL MT Sul/ Local Rio Verde de/ Faz. Alegria MT/ Col. Vanda Cunha/ Luciana F./ Data 02/IX/2004// Coleção Herman Lent/ Laboratório Nacional e/ Internacional de Referência em/ Taxonomia de Triatomíneos// CTIOC Nº 12330; Non-type material. Coleção/ Rodolfo Carcavallo// 3884 //CTIOC/ Nº. 4605 ♀; BRASIL/ Mato Grosso/ LAB/ IV-83// Coleção/ Rodolfo Carcavallo// 3880// CTIOC/ Nº. 4606 ♀; BRASIL/ Mato Grosso/ laboratório/ IV-84// 3893// Coleção/ Rodolfo Carcavallo// CTIOC/ Nº. 4610 ♂; Coleção/ Rodolfo Carcavallo// 3905// CTIOC/ Nº. 4629 ♀; Coleção/ Rodolfo Carcavallo// 3906// CTIOC/ Nº. 4630 ♀; BRASIL/ Mato Grosso/ LAB/ IV-84// Coleção/ Rodolfo Carcavallo// 3908// CTIOC/ Nº. 4643 ♀; Coleção/ Rodolfo Carcavallo// 3909// CTIOC/ Nº. 4634 ♂; *Triatoma mato-/ grossensis*/ H. LENT det./ Miranda M. Grosso/ 7-79 domic./ J. V. Alves// CTIOC/ Nº. 4636 ♀; *Triatoma mato-/ grossensis*/ H. LENT det./ Miranda M. Grosso/ 7-79 domic./ J. V. Alves// CTIOC/ Nº. 4637 ♀

Triatoma melanica Costa, Argolo & Felix, 2006

Other material
FIOCRUZ/ IOC/ *T. brasiliensis/ melanica*// BRASIL – MG/ local: Teu Espinhosa/ col: Marchen & Almeida/ & FNS 2/ Dara: 25.06.91// CEIOC/ 1538

Triatoma melanocephala Neiva & Pinto, 1923

Paratypes
Triatoma melano-/ cephala n. sp./ 8-971/ 1923/ H. Lent det.// Paraiba, zona/ do agreste/ em domicílio/ Espinola 7.971// Paratypus// Parátipo// N. 2764/ HEMIPTERA/ Inst. Oswaldo Cruz// CTIOC/ N° 9446

Other material
Mun. Arara/ Loc. Cidade/ T. melanocephala/ Data: 13.06.72/ Det D. Santos// CTIOC/ N° 7093; *T. melanocephala*//Condeúba-Ba/ Rg 85.7// 4069// Coleção/ Rodolfo Carcavallo// CTIOC/ N° 7092

Triatoma oliveirai (Neiva, Pinto & Lent, 1939)

Holotype
Eutriatoma oliveirai/ holótipo/ n° 1048// *Eutriatoma oliveirai*/ Neiva, Pinto e Lent, 1940/ Holótipo ♀/ H. LENT DET.// Porto Alegre// 34// Holotypus// HOLO/ TYPUS// N. 1048/ HEMIPTERA/ Inst. Oswaldo Cruz// CTIOC/ N° 8973

Other material
exemp 1 // Nº1049 / HEMIPTERA/ Inst. Oswaldo Cruz // *T. oliverai* // CTIOC/ Nº. 12087 ♀; *Triatoma / oliveirai* / H. LENT det. // SAPUCAIA DO SUL- RS / MORRO SAPUCAIA/ 19-X-86/ BARCELLOS, A. // 1999 // T. oliveirai ♀

Triatoma patagonica Del Ponte, 1929

Other material
Triatoma/ patagonica/ Del Ponte/ Wygodzinsky det./ INST. MED. REG.// 0099// 4399// Coleção/ Rodolfo Carcavallo// CTIOC/ N° 9279; *Triatoma/ patagonica*/ Del Ponte/ Wygodzinsky det./ INST. MED. REG.// 0099// 4400// Coleção/ Rodolfo Carcavallo// CTIOC/ N° 9280; *Triatoma/ patagonica/* Del Ponte/ Wygodzinsky det./ INST. MED. REG.// 0086// 4393// Coleção/ Rodolfo Carcavallo// CTIOC/ N° 9273; Argentina/ B. Aires/ E. Lamarca/ V-68// Coleção/ Rodolfo Carcavallo// CTIOC/ Nº 9274; Argentina/ B. Aires/ E. Tornquist/ V-68// Coleção/ Rodolfo Carcavallo// 4395// CTIOC/ Nº 9275

Triatoma petrocchiae Pinto & Barreto, 1925

Holotype
Triatoma petro-/ chii/ H. LENT det.// *Triatoma petrocchiae*/ H. LENT det.// ♂ T. petrochii/ P x Barreto R. G. Norte// Tipo// Holótipo// N. 2760/ HEMIPTERA/ Inst. Oswaldo Cruz// CTIOC/ N° 8899

Triatoma pintodiasi Jurberg, Cunha & Rocha, 2013

Holotype
Triatoma/ pintodiasi/ Det. J. Jurberg/ Data set. 2007/ N° Fiocruz 3341/ tipo ♂// FIOCRUZ/ Instituto Oswaldo Cruz/ Vila Nova do Sul/ R. G. do Sul/ Vanda Cunha/ cof. 9.06// Holotipo/ CTIOC/ N° 8900

Paratypes
Triatoma/ pintodiasi ♂/ Det José Jurberg/ Data Set. 09/ N° FIOCRUZ 3350// *T. circummaculata*/ Det. H. Lent/ Data 6.97// Rio Grande do/ Sul/ Carajava do/ Sul/ S. do Ricardinho/ 6.97// Parátipo// Parátipo/ CTIOC/ N° 8909; *Triatoma/ pintodiasi* ♂/ Det José Jurberg/ Data Set. 09/ N° FIOCRUZ 3353// Rio Grande do/ Sul/ Carajava do/ Sul/ S. do Ricardinho/ 6.97/ Parátipo/ Parátipo/ CTIOC/ N° 8912

Triatoma platensis Neiva, 1913

Other material
Triatoma platensis// Argentina/ Mendoza/ Cordoba/ v-70 // Coleção/ Rodolfo Carcavallo// 4589; Argentina/ Cordoba/ Cruz del Ele/ Laboratório/ 21-x-67// Coleção/ Rodolfo Carcavallo// 4641// CTIOC/ N° 9531; Argentina/ Cordoba/ Gral. Roca/ II-84// Coleção/ Rodolfo Carcavllo// 4648// CTIOC/ N° 9538; Arg./ La Pampa/ Rancul/ XI-83// Coleção/ Rodolfo Carcavalo// 4649// CTIOC/ N° 9539; Argentina/ La Rioja/ Ilian/ 15-I-65// Coleção/ Rodolfo Carcavallo// 4655/ CTIOC/ N° 9545

Triatoma pseudomaculata Corrêa & Espínola, 1964

Holotype
Serv. Profilax. Malaria/ São Paulo-Brasil/ *T. pseudomaculata*/ Holótipo/ F. 6885 -Sobral/ SPM-89/ Ceará// Holótipo// N. 1458/ HMIPTERA/ Inst. Oswaldo Cruz// CTIOC/ N° 8922

Other material
Brasil/ Bahia/ Lab/ X-75// Coleção/ Rodolfo Carcavallo// 4889// CTIOC/ N° 4649; Brasi/ M. Gerais/ Lab/ X-75// Coleção/ Rodolfo Carcallo// 4887// CTIOC/ N° 4647; Brasil-San Salvador/ Bahia- Lab. Inaiú/ T. pseudomaculata/ 3-I-68 – 1725:4// 3767// Coleção/ Rodolfo Carcavallo// CTIOC/ N° 4660; Brasil/ Rio G do/ N Lab/ X-75// Coleção/ Rodolfo Carcavallo// 4890// CTIOC/ N° 4650; Brasil/ Ceará/ Lab/ X-75// Coleção/ Rodolfo Carcavallo/ 4885// CTIOC/ N° 4645

Triatoma rosai Alevi, Oliveira, Garcia, Cristal, Delgado, Bittinelli, Reis, Ravazi, Oliveira, Galvão, Azeredo-Oliveira & Madeira, 2020

Paratypes
Triatoma rosai Alevi,/ Garcia, Oliveira et/ al. 2020// ARGENTINA, Department/ San Miguel, Province of/ Corrientes, 09.08.1999,/ collection manual//Paratype// CTIOC/ N° 20586; *Triatoma rosai* Alevi,/ Garcia, Oliveira et/ al. 2020// ARGENTINA, Department/ San Miguel, Province of/ Corrientes, 09.08.1999,/ collection manual//Paratype// CTIOC/ N° 20587

Triatoma rubrofasciata (De Geer, 1773)

Other material
Triatoma ru-/ brofasciata/ (De Geer)/ R. Carcavallo Det. 2000// *Triatoma rubrof-/ asciata* (De Geer)/ Det. W. Cai 1996-III/ Nanning/ Guangxi/ China/ 1982-V-23// CTIOC/ N° 9863; *Triatoma ru-/ brofasciata/* (De Geer)/ R. Carcavallo det. 2000// Tianlin/ Guagxi/ China/ 1982-v-23// CTIOC/ N° 9864; *Triatoma ru-/ brofasciata/* (De Geer)/ R. Carcavallo det. 2000// Fangcheng/ Guangxi/ China/ 1991-VII-5// CTIOC/ N° 9865

Triatoma rubrovaria (Blanchard, 1843)

Other material
Eutriatoma rubrovaria/ 1939/ H. LENT DET.// CTIOC/ N° 9947; Dom Pedrito/ R. G. Sul. I-64/ S. J. Oliveira// CTIOC N° 4997

Triatoma sherlocki Papa, Jurberg, Carcavallo, Cequeira & Barata, 2002

Paratypes
Triatoma sherlocki sp. n. Papa,/ Jurberg, Carcavallo, Cerqueira/ & Barata 2002// Santo Inácio, BA/ Brasil/ Col. Cerqueira 1974// PARÁTIPO// Parátipo// RCT 162// N° FIOCRUZ 5662// CTIOC/ N° 9763; Triatoma sherlocki sp. n. Papa,/ Jurberg, Carcavallo, Cerqueira/ & Barata 2002// Santo Inácio, BA/ Brasil/ Col. Cerqueira 1974// PARÁTIPO// Parátipo// RCT 162// N° FIOCRUZ 5664// CTIOC/ N° 9765; Triatoma sherlocki sp. n. Papa,/ Jurberg, Carcavallo, Cerqueira/ & Barata 2002// Santo Inácio, BA/ Brasil/ Col. Cerqueira 1974// PARÁTIPO// Parátipo// RCT 162// N° FIOCRUZ 5665// CTIOC/ N° 9831

Triatoma sordida (Stål, 1859)

Other material
T. sordida G2/ Det. F. Noireau/ Data VII-97// Tita/ Way to Izozog (Chaco)/ Bolivia 10.96/ F. Noireau// Cdca 80//CTIOC/ N° 9866; T. sordida G1/ Det. F. Noireau/ Data VII-97// Tita/ Way to Izozog (Chaco)/ Bolivia 10.96/ F. Noireau// Cdca 104//CTIOC/ N° 9867; T. sordida G2/ Det. F. Noireau/ Data VII-97// Tita/ Way to Izozog (Chaco)/ Bolivia 10.96/ F. Noireau// Cdca 104// CTIOC/ N° 9868;

Triatoma vandae Carcavallo, Jurberg, Rocha, Galvão, Noireau & Lent, 2002

Holotype
Triatoma/ vandae/ n° 5635// Triatoma/ vandae/ sp. n./ R. Carcavallo det. 2002// HOLOTIPO// CTIOC/ N° 9832

Paratypes
Triatoma/ vandae/ sp. n./ R. Carcavallo det. 2002// Triatoma/ vandae/ nº 5636// Parátipo// ALOTIPO// CTIOC/ N° 9833

Triatoma vitticeps (Stål, 1859)

Syntype
Vitticeps/ Stål // Rio v. Lgsdf// Type// 2930 [analyzed by photographs, Hemimetabola Collection, Museum für Naturkunde, Berlin, Germany]

Other material
Brasil/ M. Gerais/ Lab// Coleção/ Rodolfo Carcavallo// 5299// CTIOC/ N° 10063; Brasil/ M. Gerais// Coleção/ Rodolfo Carcavallo// CTIOC/ N° 10058; Triatoma vitticeps/ (Stal)/ H. LENT DET.// Reared in laboratory/ by H. Lent// Rio de Janeiro, Brasil// Coleção/ Rodolfo Carcavallo// 5301// CTIOC/ N° 10065

Triatoma williami Galvão, Souza & lima, 1965

Paratype
T. williami/ Galvão e col./ 1965 ♀/ M. Piranhas/ E. Goiás// 2353// BUTANTAN// 14// Paratypus; T. williami/ Galvão & Col., 1965 v/ M. Piranhas/ E, Goiás// Paratypus// Parátipo// Parátipo 186// CTIOC/ N° 13216

Allotype
T. williami/ Galvão e col./ 1965 ♂/ M. Piranhas/ E. Goiás// 186// BUTANTAN// Paratypus// parátipo// Nº 1762/ HEMIPTERA/ Inst. Oswaldo Cruz; T. williami/ Galvão e col./ 1965 ♂?/ Piranhas, Goiás/ A. Galvão del. 1969// 870// 37/ Alótipo ♂

Triatoma wygodzinskyi Lent, 1951

Paratypes
Triatoma wygodzinskyi/ parátipo/ Nº 1174// Sta. Rita de/ Caldas, Minas/ A. V. Martins/ 1-947// PARA/ TYPUS// N° 1174/ HEMIPTERA/ Inst. Oswaldo Cruz// Parátipo// CTIOC/ N° 9450; wygodzinskyi// Sta. Rita de/ Caldas, Minas/ A. V. Martins/ 194// PARA/ TYPUS// N° 1175/ HEMIPTERA/ Inst. Oswaldo Cruz// Parátipo// CTIOC/ N° 8924;

Allotype
Triatoma wygod/ zinskyi Lent/ H. LENT det.// Sta. Rita de/ Caldas, Minas/ A. V. Martins/ 1947// ALLO/ TYPUS// N° 1172/ HEMIPTERA/ Inst. Oswaldo Cruz// CTIOC/ N° 8923

Outgroup

Dipetalogaster maxima (Uhler, 1804)

Other material
El Triunfo// n°167// CTIOC 12505; El Triunfo// n°168// CTIOC 12506; El Triunfo// n°186// CTIOC 12504; Mexico/ Baja Calf. S./ Lab./ I-74// Coleção/ Rodolfo Carcavallo// 1472// CTIOC/ N° 2672; Mexico/ Baja Calf. S./ Lab./ V-74// Coleção/ Rodolfo Carcavallo// 1513// CTIOC/ N° 2713

Eratyrus mucronatus Stål, 1859

Syntype
Typus // Mucronatus / N. / Demer. Wust // Eratyrus / Stål // "mucronatus / Stål // "2914" [Hemimetabola Collection, Museum für Naturkunde, Berlin, Germany]

Other material
Brasil – Amazonas/ Manaus/ Reserva Ducke/ 20.8.68/
R Kasso col.// N. 1967/ HEMIPTERA/ Inst. Oswaldo Cruz// CTIOC/ N° 2787; Eratyrus ♂/ mucronatus/ Utinga Belém/ 26.9.77 mamiles/ N° 102/ IOC/ 2644// CTIOC N° 2785

Nesotriatoma bruneri Usinger, 1944

Other material
Triatoma bruneri/ (Usinger)/ 1981 H. LENT det.// Cuba, Pireas/ del Rio 11-979/ Cova de animais// Coleção Herman Lent/ Laboratório Nacional e/ Internacional de Referência em/ Taxonomia de Triatomíneos// 6// 2005// CTIOC/ N° 2880; T. bruneri/ loc. Granahacabibes/ Cuba la sorda. / 1984// Coleção/ Rodolfo Carcavallo// 1845// CTIOC/ N° 2848; T. bruneri/ loc. Granahacabibes/ Cuba of Frances. / 1984// Coleção/ Rodolfo Carcavallo// 1846// CTIOC/ N° 2849; Nesotriatoma bruneri/ Usinger, 1944// Cuba// 98// CTIOC/ N° 12456; Nesotriatoma bruneri/ Usinger, 1944// Cuba// 99// CTIOC/ Nº 12457

Panstrongylus megistus (Burmeister, 1835)

Syntype
Type // Bras. s. am // Conorh. / megistus / Br. // 2937 [analyzed by photographs, Hemimetabola Collection, Museum für Naturkunde, Berlin, Germany]

Other material
128 // CTIOC 12386; Leg glued to paper// São Paulo, /Juquiá, Faz. Poço Grande,/ 6-9.IV.1940 – F. Lane &/ Trav. Fo. & C. Carvallho.// N. 690/ HEMIPTERA/ Inst. Oswaldo Cruz // CTIOC/ N° 1623; BRASIL/ -S. Paulo/ Boracea 900 ms/ 13.vi.1942/ Almeida & Trav. Fº// N. 684/ HEMIPTERA/ Inst. Oswaldo Cruz; CTIOC/ N° 1617; BRASIL - São

Panstrongylus rufotuberculatus (Champion, 1899)

Other material
Panstrongylus/ rufotuberculatus/ n° 5619// Guayacan,/ Equador/ Col. Garcia-Zepata/ III-1999// CTIOC N° 1780; P. rufotuberculatus Dom./ Carrasco La Paz 6.93// 3023// CTIOC/ N° 1423

Panstrongylus tibiamaculatus (Pinto, 1926)

Syntype
Eutriatoma tibiamaculata/ Pinto 1920/ Typo//Angra dos Reis/ L T. 16.1926

Other material
Eutriatoma tibia-/ maculata Pinto, 1926/ XI-940/ H. LENT. DET.// Juquiá – S. Paulo/ A' Cruz – 3.XI.940/ Trav. Trav. Coll.// CTIOC/ N° 2884; Eutriatoma tibia-/ maculata Pinto, 1926/ ♀ XII-934/ H. LENT DET.// Juquiá – S. Paulo/ Francisco Lane/ col./ XII-939// desenhado// CTIOC/ N° 2885; Triatoma/ tibiamaculata/ (Pinto)/ Wygodzinsky det./ INST. MED. REG.// Corupã/ St. Catarina/ XII-1949/ Malles// CTIOC/ N° 2886;

Panstrongylus tupynambai Lent, 1942

Holotype
Panstrongylus tupy-/ nambai Lent, 1942/ ♀ Holótipo/ H. LENT DET.// Munic. Caçapava; Rio G. Sul/ Beltrão & Tupynam-/ bá 19.1.942// HOLOTYPUS// Holótipo// N° 730/ HEMIPTERA Inst. Oswaldo Cruz// CTIOC/ N° 1747

Allotype
Panstrongylus tupy-/ nambai Lent, 1942/ ♂ Alótipo/ H. LENT DET.// Munic. Caçapava; Rio G. Sul/ Beltrão & Tupynam-/ bá 19.1.942// ALLOTYPUS// N° 731/ HEMIPTERA Inst. Oswaldo Cruz// CTIOC/ N° 1752

Other material
Panstrongylus/ tupynambai Lent,/ 1942// Brasil/ Sítio Faxina – Pinheiro/ Machado - RS// 138// LAEN.RS// 032674// CTIOC N° 12494

Psammolestes tertius Lent & Jurberg, 1965

Paratype
Carmo Rio Claro/ M. Gerais/ J. Candido col/ 6. 948// Parátipo// Paratypus// N. 2303/ HEMIPTERA/ Inst. Oswaldo Cruz// CTIOC/ N° 13255

Other material
Psammolestes / tertius Lent / e Jurberg, / 1965 // Ninhos Phacellodomus / rufiprons / Singitalis cas // I.O.Cruz – Brasil/ Mato Grosso, Salobra,/ 1-9.III.1940 // Coleção Herman Lent/ Laboratório Nacional e/ Internacional de Referência em/ Taxonomia de Triatomíneos // N. 2199/ HEMIPTERA/ Inst. Oswaldo Cruz // CTIOC N° 11212; Psammolestes/ tertius Lent/ e Jurberg,/ 1965// Ninhos Phacellodomus / rufiprons / Singitalis cas // I.O.Cruz – Brasil/ Mato Grosso, Salobra,/ 1-9.III.1940 // Coleção Herman Lent/ Laboratório Nacional e/ Internacional de Referência em/ Taxonomia de Triatomíneos // N. 2200/ HEMIPTERA/ Inst. Oswaldo Cruz // CTIOC N° 11213; Psammolestes / tertius Lent / e Jurberg, / 1965 // Ninhos Phacellodomus / rufiprons / Singitalis cas // I.O.Cruz – Brasil/ Mato Grosso, Salobra,/ 1-9.III.1940 // Coleção Herman Lent/ Laboratório Nacional e/ Internacional de Referência em/ Taxonomia de Triatomíneos // N. 2197/ HEMIPTERA/ Inst. Oswaldo Cruz // CTIOC N° 11210; Psammolestes / tertius Lent / e Jurberg, / 1965 // Ninhos Phacellodomus / rufiprons / Singitalis cas // I.O.Cruz – Brasil/ Mato Grosso, Salobra,/ 1-9.III.1940 // Coleção Herman Lent/ Laboratório Nacional e/ Internacional de Referência em/ Taxonomia de Triatomíneos // N. 2198/ HEMIPTERA/ Inst. Oswaldo Cruz// CTIOC N° 11211

Rhodnius stali Lent, Jurberg & Galvão, 1993

Holotype
Rhodnius stali/ Lent, Jurberg/ e Galvão/ 1993// Rhodnius pictipes/ Stali, 1872/ 3/947 H. LENT DET.// desenhado// Holotipo// N° 645/ HEMIPTERA/ Inst. Oswaldo Cruz// CTIOC/ N° 8919
Other material
R. stali// 05

Triatoma phyllosoma (Burmeister, 1835)

Syntype
phyllo-/ soma/ Br.// Mexico Deppe// Typus/ 2918 [analyzed by photographs, Hemimetabola Collection, Museum für Naturkunde, Berlin, Germany]

Other material
Triatoma phyllosoma/ criação laboratório/ México 10.934/ H. LENT DET.; *T. phyllosoma/ usingeri* Moaz; *T. phyllosoma/ usingeri* Moaz1; *T. phyllosoma/ usingeri* Moaz1// Reared in laboratory/ by H. Lent/ Rio de Janeiro, Brasil; *T. phyllosoma*/ Mexico, 1938/ Caballero leg/ H. LENT DET.// CTIOC/ N° 2904; *Triatoma phyllo-/ soma* ♀/ Mexico-X-938/ H. LENT DET.// CTIOC/ N° 2908

Data 2. Measurements and indices.

Hl Head length. In dorsal view, imaginary line drawn longitudinally between the anterior margin of the clypeus and the margin of the postocular region that precedes the neck [Supplementary data (Fig. 1.1)].
Ew Eye width. In dorsal view, imaginary line drawn transversely in the median region, between the outer and inner margins of the right eye [Supplementary data (Fig. 1.1)].
La Length of the anteocular region. In dorsal view, imaginary line drawn longitudinally, between the margin of the clypeus and the anterior margin of the eyes [Supplementary data (Fig. 1.1)].
Lp Length of the postocular region. In dorsal view, imaginary line drawn longitudinally, between the posterior margin of the eye and the posterior margin of the head, anterior to the neck [Supplementary data (Fig. 1.1)].
Wh Maximum width of the head between the eyes [across eyes]. In dorsal view, imaginary line drawn transversally between the outer margins of each eye [Supplementary data (Fig. 1.2)].
Wi Width of the interocular space. In dorsal view, imaginary line drawn transversely in the median region, the smallest distance between the inner margins of the eyes [Supplementary data (Fig. 1.2)].
Pl Pronotum length: imaginary line drawn longitudinally in the median region of the structure, between the proximal and distal margins [Supplementary data (Fig. 1.3)].
Ls Length of the scutellum (main body). In dorsal view, imaginary line plot drawn longitudinally in the median region, between the anterior margins and apex of the scutellar process [Supplementary data (Fig. 1.3)].
Lp Length of the scutellum process. In dorsal view, imaginary line drawn longitudinally in the median region, between the margin posterior part of the body of the scutellum and the apex of the scutellar process [Supplementary data (Fig. 1.3)].

TABLE I

Data matrix

	**1**	**2**	**3**	**4**	**5**	**6**	**7**	**8**	**9**	**10**	**11**	**12**	**13**	**14**	**15**	**16**	**17**	**18**	**19**	**20**	**21**	**22**	**23**	**24**	**25**
Rhodnius stali	0	0	0	0	0	0	0	0	0	0	0	0	0	0	0	0	0	0	0	0	0	0	0	0	0
Dipetalogaster maxima	0	0	1	-	0	1	1	-	3	0	8	1	1	-	1	-	3	0	1	1	1	0	1	0	1
Eratyrus mucronatus	0	0	1	-	0	1	1	-	3	1	-	1	1	-	1	-	0	1	1	1	1	0	1	0	0
Triatoma phyllosoma	2	0	1	-	0	1	1	-	3	0	4	0	1	-	1	-	3	1	1	1	1	0	1	0	0
Nesotriatoma bruneri	0	0	0	1	0	1	1	-	3	0	0	1	1	-	1	-	3	1	1	1	1	0	1	0	0
Panstrongylus megistus	0	1	1	-	0	1	1	-	3	0	4	1	1	-	1	-	2	2	1	1	1	1	1	1	1
Panstrongylus rufotuberculatus	1	0	0	1	0	1	1	-	3	0	7	1	0	1	1	-	1	2	1	1	1	1	1	1	1
Panstrongylus tibiamaculatus	0	0	1	-	0	1	1	-	3	0	6	1	1	-	0	1	1	1	1	1	1	0	1	0	0
Panstrongylus tupynambai	0	0	1	-	1	-	1	-	3	0	6	1	1	-	1	-	2	2	1	1	1	1	1	1	1
Psammolestes tertius	0	0	0	2	0	2	0	2	0	0	4	0	0	4	0	2	0	0	2	0	1	2	1	1	1
Triatoma arthurneivai	0	0	1	-	0	1	1	-	3	0	3	1	1	-	1	-	3	1	1	1	1	0	1	1	0
Triatoma bahiensis	0	1	1	-	0	1	1	-	3	0	3	1	1	-	1	-	1	0	1	1	1	0	1	0	0
Triatoma baratai	0	0	1	-	0	1	1	-	3	0	8	1	1	-	1	-	1	0	1	1	1	0	1	0	0
Triatoma brasiliensis	0	1	1	-	0	1	0	1	3	0	3	1	0	2	1	-	3	1	1	1	1	0	1	0	0
Triatoma carcavalloi	0	0	1	-	0	1	1	-	2	0	2	1	1	-	1	-	3	1	1	1	1	0	1	0	0
Triatoma circummaculata	0	0	1	-	0	1	1	-	2	0	2	1	1	-	1	-	3	1	1	1	1	0	1	0	0
Triatoma costalimai	0	0	1	-	0	1	1	-	3	0	3	1	1	-	1	-	3	1	1	1	1	0	1	0	0
Triatoma deaneorum	0	0	1	-	0	1	1	-	3	0	1	1	0	3	1	-	3	1	1	1	1	0	1	1	0
Triatoma delpontei	0	1	1	-	0	1	1	-	3	0	4	1	1	-	1	-	3	1	1	1	1	0	1	1	0
Triatoma garciabesi	0	0	1	-	0	1	1	-	0	0	1	1	0	3	1	-	1	0	1	0	1	0	1	1	0
Triatoma guasayana	0	0	1	-	0	1	1	-	3	0	1	1	0	3	0	4	3	0	1	1	1	0	1	1	0
Triatoma infestans	0	1	1	-	0	1	1	-	3	0	3	1	1	-	1	-	3	1	1	1	1	0	1	0	0
Triatoma jatai	0	0	1	-	0	1	1	-	0	0	3	1	1	-	1	-	3	1	1	1	1	0	1	0	0
Triatoma juazeirensis	0	1	1	-	0	1	1	-	3	0	3	1	1	-	1	-	3	1	1	1	1	0	1	0	0
Triatoma jurbergi	0	0	1	-	0	1	1	-	0	0	3	1	1	-	1	-	1	1	1	1	1	0	1	0	0
Triatoma klugi	0	0	1	-	0	1	1	-	3	0	3	1	1	-	1	-	3	1	1	1	1	0	1	0	0
Triatoma lenti	0	1	1	-	0	1	1	-	3	0	3	1	1	-	1	-	3	1	1	1	1	0	1	0	0
Triatoma limai	0	0	1	-	0	1	1	-	3	0	3	1	1	-	1	-	3	1	1	1	1	0	1	0	0
Triatoma maculata	0	0	0	1	0	1	1	-	3	0	3	1	1	-	1	-	3	1	1	1	1	0	1	1	0
Triatoma matogrossensis	0	0	1	-	0	1	1	-	3	0	2	1	0	3	1	-	3	1	1	1	1	0	1	0	0
Triatoma melanica	0	1	1	-	0	1	1	-	3	0	3	1	0	-	0	3	3	0	1	1	1	0	1	0	0
Triatoma melanocephala	0	1	1	-	0	1	1	-	3	0	3	1	1	-	1	-	3	1	1	1	1	0	1	1	0
Triatoma infestans var. melanosoma	0	1	1	-	1	-	1	-	3	0	5	1	1	-	1	-	3	1	1	1	1	0	1	0	0
Triatoma oliveirai	0	0	1	-	0	1	1	-	3	0	3	1	1	-	1	-	3	1	1	1	1	0	1	0	0
Triatoma patagonica	0	0	1	-	0	1	1	-	3	0	3	1	0	1	1	-	3	0	1	1	1	0	1	0	0
Triatoma petrocchiae	0	0	1	-	0	1	0	1	3	0	3	1	1	-	1	-	3	1	1	1	1	0	1	0	0
Triatoma pintodiasi	0	0	1	-	0	1	1	-	1	0	2	1	1	-	1	-	3	1	1	1	1	0	1	0	0
Triatoma platensis	2	1	1	-	0	1	1	-	0	0	4	1	1	-	1	-	3	1	1	1	1	0	1	1	1
Triatoma pseudomaculata	0	0	1	-	0	1	1	-	3	0	3	1	1	-	1	-	3	1	1	1	1	0	1	1	0
Triatoma rosai	0	0	1	-	0	1	1	-	0	0	1	1	1	3	1	-	1	0	1	0	1	0	1	1	0
Triatoma rubrovaria	0	0	1	-	0	1	1	-	3	0	3	1	1	-	1	-	3	1	1	1	1	0	1	0	0
Triatoma rubrofasciata	0	1	1	-	0	3	0	1	0	0	5	1	1	-	1	-	4	0	0	0	1	0	1	1	1
Triatoma sherlocki	0	1	1	-	0	1	1	-	3	0	3	1	0	2	1	-	3	1	1	1	1	0	1	0	0
Triatoma sordida	0	0	1	-	0	1	1	-	0	0	1	1	0	3	1	-	1	0	1	0	1	0	1	1	0
Triatoma vandae	0	0	1	-	0	1	1	-	3	0	2	1	0	3	1	-	3	1	1	1	1	0	1	0	0
Triatoma vitticeps	0	1	0	1	0	1	1	-	3	0	3	1	1	-	1	-	3	1	1	1	1	0	1	1	1
Triatoma williami	0	0	1	-	0	1	1	-	3	0	2	1	1	-	1	-	3	1	1	1	1	0	1	0	0
Triatoma wygodzinskyi	0	0	1	-	0	1	1	-	3	0	3	1	1	-	1	-	3	1	1	1	1	0	1	1	0

	26	27	28	29	30	31	32	33	34	35	36	37	38	39	40	41	42	43	44	45	46	47	48	49	50
Rhodnius stali	0	0	0	0	0	0	0	0	0	0	0	0	0	0	0	0	0	0	0	-	0	0	0	0	0
Dipetalogaster maxima	0	2	1	1	1	1	0	0	0	0	1	0	1	0	1	0	1	1	0	-	0	1	1	1	0
Eratyrus mucronatus	0	1	1	1	0	0	0	0	0	0	0	0	1	0	1	0	0	0	0	-	1	1	0	0	0
Triatoma phyllosoma	0	1	1	1	1	2	0	0	0	0	0	0	0	0	1	0	1	1	0	-	1	1	0	0	1
Nesotriatoma bruneri	0	1	1	1	1	1	0	1	0	0	0	0	1	0	1	1	0	0	0	-	1	1	0	0	1
Panstrongylus megistus	0	1	1	1	2	1	0	0	0	0	0	0	0	0	2	0	0	1	0	-	1	1	0	1	1
Panstrongylus rufotuberculatus	0	2	1	1	1	1	0	1	0	1	0	1	1	0	2	0	0	1	0	-	1	1	0	0	1
Panstrongylus tibiamaculatus	0	1	1	0	2	1	0	1	0	0	0	1	1	0	1	0	0	0	1	1	1	1	0	0	1
Panstrongylus tupynambai	1	1	1	1	1	2	0	0	0	0	0	1	0	0	2	0	0	1	0	-	0	1	0	1	1
Psammolestes tertius	1	2	0	0	0	0	0	0	0	1	0	0	1	1	0	1	0	1	1	0	0	1	0	0	0
Triatoma arthurneivai	1	1	1	0	1	1	0	1	0	0	0	0	1	0	1	1	0	0	0	-	0	1	0	0	0
Triatoma bahiensis	2	1	1	0	1	1	0	1	1	1	1	0	1	0	1	0	0	1	0	-	0	1	0	0	0
Triatoma baratai	2	1	1	0	1	1	0	1	0	0	0	0	1	0	1	1	0	0	0	-	0	1	0	0	0
Triatoma brasiliensis	2	1	1	0	1	1	0	1	0	1	1	0	1	0	1	0	0	0	0	-	0	1	0	0	0
Triatoma carcavalloi	1	1	1	0	1	1	0	1	0	0	0	0	1	0	1	1	0	0	0	-	0	1	1	0	0
Triatoma circummaculata	1	2	1	0	1	1	1	1	2	0	0	0	1	0	1	1	1	1	0	-	0	1	1	0	0
Triatoma costalimai	2	1	1	0	1	1	0	1	0	0	1	0	1	0	1	1	0	0	0	-	0	1	0	0	0
Triatoma deaneorum	2	1	1	0	1	1	0	1	0	0	0	0	1	0	1	1	-	0	0	-	0	1	0	0	1
Triatoma delpontei	0	1	1	0	2	2	0	1	0	1	1	0	1	0	1	1	0	0	0	-	0	1	1	1	1
Triatoma garciabesi	0	1	1	0	1	1	0	1	0	0	0	0	1	0	1	1	0	0	0	-	0	1	1	1	1
Triatoma guasayana	0	1	1	0	1	1	0	1	1	0	0	0	1	0	1	1	0	0	0	-	0	1	1	0	1
Triatoma infestans	0	1	1	0	1	1	0	1	0	1	0	0	1	0	1	1	0	0	0	-	0	1	1	1	1
Triatoma jatai	2	1	1	0	1	1	0	1	0	0	1	0	1	0	1	1	0	0	0	-	0	1	0	0	0
Triatoma juazeirensis	2	1	1	0	1	1	0	1	0	1	1	0	1	0	1	0	0	0	0	-	0	1	0	0	0
Triatoma jurbergi	3	1	1	0	1	1	0	1	0	0	0	0	1	0	1	1	0	0	0	-	0	1	0	0	0
Triatoma klugi	0	1	1	0	1	1	0	1	0	0	0	0	1	0	1	1	0	0	0	-	0	1	1	0	0
Triatoma lenti	2	1	1	0	1	1	0	1	0	1	1	0	1	0	1	0	0	0	0	-	0	1	0	0	0
Triatoma limai	1	2	1	0	1	1	1	1	2	0	0	0	1	0	1	1	0	1	0	-	0	1	1	0	0
Triatoma maculata	0	1	1	0	1	1	0	1	0	0	0	0	0	0	1	1	0	0	0	-	1	1	0	1	1
Triatoma matogrossensis	2	1	1	0	1	1	0	1	0	0	1	0	1	0	1	1	0	0	0	-	0	1	0	0	0
Triatoma melanica	2	1	1	0	1	1	0	1	0	1	1	0	1	0	1	0	0	0	0	-	0	1	0	0	0
Triatoma melanocephala	0	1	1	0	1	1	0	1	0	0	0	0	1	0	1	0	0	0	0	-	1	1	0	0	1
Triatoma infestans var. melanosoma	2	1	1	0	1	1	0	1	0	1	0	0	1	0	1	1	0	0	0	-	0	1	1	1	0
Triatoma oliveirai	0	1	1	0	1	1	0	1	0	0	1	0	1	0	1	1	0	1	0	-	0	1	1	0	0
Triatoma patagonica	1	1	1	0	1	1	0	1	0	0	0	0	1	0	1	1	0	0	0	-	0	1	0	1	0
Triatoma petrocchiae	2	1	1	0	1	1	0	1	0	1	1	0	1	0	1	1	0	0	0	-	0	1	0	0	0
Triatoma pintodiasi	1	1	1	0	1	1	1	1	1	0	0	0	1	0	1	1	0	1	0	-	0	1	1	0	0
Triatoma platensis	0	1	1	0	1	2	0	1	0	1	0	0	1	0	1	1	0	0	0	-	0	1	1	1	1
Triatoma pseudomaculata	0	1	1	0	1	1	0	1	0	0	0	0	1	0	1	1	0	0	0	-	0	1	0	0	1
Triatoma rosai	0	1	1	0	1	1	0	1	0	0	0	0	1	0	1	1	0	0	0	-	0	1	1	1	1
Triatoma rubrovaria	0	1	1	0	1	1	0	1	0	0	0	0	1	0	1	1	0	0	0	-	0	1	1	0	0
Triatoma rubrofasciata	1	1	1	0	1	1	0	0	0	1	0	0	0	0	1	1	0	1	1	0	0	1	0	0	0
Triatoma sherlocki	3	1	1	0	1	1	0	1	0	1	1	0	1	0	1	1	0	0	0	-	0	1	0	0	0
Triatoma sordida	0	1	1	0	1	1	0	1	0	0	0	0	1	0	1	1	0	0	0	-	0	1	1	1	1
Triatoma vandae	2	1	1	0	1	1	0	1	0	0	0	0	1	0	1	1	0	0	0	-	0	1	0	0	0
Triatoma vitticeps	0	1	1	0	2	2	0	1	0	0	0	0	1	0	1	0	0	0	0	-	1	1	0	0	1
Triatoma williami	2	1	1	0	1	1	0	1	0	0	0	0	1	0	1	1	0	0	0	-	0	1	0	0	0
Triatoma wygodzinskyi	1	1	1	0	1	1	0	1	0	0	0	0	1	0	1	1	0	0	0	-	0	1	0	0	0

	51	52	53	54	55	56	57	58	59	60	61	62	63	64	65	66	67	68	69	70	71	72
Rhodnius stali	0	0	0	0	0	0	0	0	0	0	0	0	0	0	0	0	0	0	0	0	0	0
Dipetalogaster maxima	0	0	0	1	1	1	0	0	0	0	0	0	1	0	2	1	1	1	1	1	1	2
Eratyrus mucronatus	0	1	0	1	1	0	1	1	2	0	0	0	1	0	1	0	0	0	1	1	0	1
Triatoma phyllosoma	0	0	0	1	1	0	2	0	0	0	0	1	1	1	1	0	0	0	1	1	1	2
Nesotriatoma bruneri	1	0	1	0	0	0	2	0	0	0	0	0	1	0	1	0	0	0	1	1	1	2
Panstrongylus megistus	0	0	0	1	0	1	0	2	1	0	0	0	1	0	1	0	0	0	1	1	0	1
Panstrongylus rufotuberculatus	0	0	0	1	0	1	0	2	0	0	1	0	1	0	1	0	0	0	1	1	0	1
Panstrongylus tibiamaculatus	0	0	0	1	1	0	0	0	0	1	1	0	1	0	1	0	0	0	1	1	0	0
Panstrongylus tupynambai	0	0	0	1	0	0	2	0	0	0	0	0	1	0	1	0	0	0	1	1	0	1
Psammolestes tertius	0	0	0	0	0	1	0	0	0	1	1	0	0	0	0	0	0	0	0	0	0	0
Triatoma arthurneivai	0	0	0	1	1	0	2	0	0	1	1	0	1	0	1	0	0	0	1	1	0	1
Triatoma bahiensis	0	0	0	1	1	0	2	0	0	1	1	0	1	0	1	0	0	0	1	1	0	0
Triatoma baratai	0	0	0	1	1	1	0	0	0	1	1	0	1	0	1	0	0	0	1	1	0	1
Triatoma brasiliensis	0	0	0	1	1	0	2	0	0	1	1	0	1	0	1	0	0	0	1	1	0	1
Triatoma carcavalloi	0	0	0	1	1	1	0	0	0	1	1	0	1	0	1	0	0	0	1	1	0	1
Triatoma circummaculata	0	0	0	1	1	1	0	0	0	1	1	0	1	0	1	0	0	0	1	1	0	1
Triatoma costalimai	0	0	0	1	1	0	2	0	0	1	1	0	1	0	1	0	0	0	1	1	0	1
Triatoma deaneorum	0	0	0	1	1	0	0	0	0	1	1	0	1	0	1	0	0	0	1	1	0	1
Triatoma delpontei	0	0	0	1	1	1	0	0	0	1	1	0	1	0	1	0	0	0	1	1	0	1
Triatoma garciabesi	0	0	0	1	1	0	2	0	0	1	1	0	1	0	1	0	0	0	1	1	0	1
Triatoma guasayana	0	0	0	1	1	1	2	0	0	1	1	0	1	0	1	0	0	0	1	1	0	1
Triatoma infestans	0	0	0	1	1	0	0	0	0	1	1	0	1	0	1	0	0	0	1	1	0	1
Triatoma jatai	0	0	0	1	1	0	2	0	0	1	1	0	1	0	1	0	0	0	1	1	0	1
Triatoma juazeirensis	0	0	0	1	1	0	2	0	0	1	1	0	1	0	1	0	0	0	1	1	0	1
Triatoma jurbergi	0	0	0	1	1	1	0	0	0	1	1	0	1	0	1	0	0	0	1	1	0	1
Triatoma klugi	0	0	0	1	1	1	0	0	0	1	1	0	1	0	1	0	0	0	1	1	0	1
Triatoma lenti	0	0	0	1	1	0	2	0	0	1	1	0	1	0	1	0	0	0	1	1	0	1
Triatoma limai	0	0	0	1	1	1	2	0	0	1	1	0	1	0	1	0	0	0	1	1	0	1
Triatoma maculata	0	0	0	1	1	0	2	0	0	1	1	0	1	0	1	0	0	0	1	1	0	1
Triatoma matogrossensis	0	0	0	1	1	1	0	0	0	1	1	0	1	0	1	0	0	0	1	1	0	1
Triatoma melanica	0	0	0	1	1	0	2	0	0	1	1	0	1	0	1	0	0	0	1	1	0	1
Triatoma melanocephala	0	0	0	1	1	0	2	0	0	1	1	0	1	0	1	0	0	0	1	1	0	1
Triatoma infestans var. melanosoma	0	0	0	1	1	0	0	0	0	1	1	0	1	0	1	0	0	0	1	1	0	1
Triatoma oliveirai	0	0	0	1	1	1	0	0	0	1	1	0	1	0	1	0	0	0	1	1	1	?
Triatoma patagonica	0	0	0	1	1	0	2	0	0	1	1	0	1	0	1	0	0	0	1	1	0	1
Triatoma petrocchiae	0	0	0	1	0	0	2	0	0	1	1	0	1	0	1	0	0	0	1	1	1	2
Triatoma pintodiasi	0	0	0	1	1	1	0	0	0	1	1	0	1	0	1	0	0	0	1	1	0	1
Triatoma platensis	0	0	0	1	1	0	0	0	0	1	1	0	1	0	1	0	0	0	1	1	0	1
Triatoma pseudomaculata	0	0	0	1	1	0	2	0	0	1	1	0	1	0	1	0	0	0	1	1	0	1
Triatoma rosai	0	0	0	1	1	1	2	0	0	1	1	0	1	0	1	0	0	0	1	1	0	1
Triatoma rubrovaria	0	0	0	1	1	1	2	0	0	1	1	0	1	0	1	0	0	0	1	1	0	1
Triatoma rubrofasciata	1	0	1	1	0	1	0	0	0	1	1	0	1	0	1	0	0	0	1	1	0	1
Triatoma sherlocki	0	0	0	1	1	0	0	0	0	1	1	1	1	0	1	0	0	0	1	1	1	2
Triatoma sordida	0	0	0	1	1	0	2	0	0	1	1	0	1	0	1	0	0	0	1	1	0	1
Triatoma vandae	0	0	0	1	1	1	0	0	0	1	1	0	1	0	1	0	0	0	1	1	0	1
Triatoma vitticeps	0	0	0	1	0	0	2	0	0	1	1	0	1	0	1	0	0	0	1	1	0	0
Triatoma williami	0	0	0	1	1	1	0	0	0	1	1	0	1	0	1	0	0	0	1	1	0	1
Triatoma wygodzinskyi	0	0	0	1	1	0	2	0	0	1	1	0	1	0	1	0	0	0	1	1	0	1

TABLE II

List of characters

	Characters and states	L	CI	RI
1	Body surface, setae: (0) Short and sparse or barely visible [Supplementary data (Fig. 3.1)]; (1) Short and dense [Supplementary data (Fig. 3.2)]; (2) Long and dense [Supplementary data (Fig. 3.3)]	3	66	0
2	Labium, segments III and IV (second and third visible segments, respectively), setae, in lateral view: (0) Short [Supplementary data (Fig. 3.4)]; (1) Long [Supplementary data (Fig. 3.5)]	5	20	66
3	Head, spots, in dorsal view: (0) Present [Supplementary data (Fig. 3.2)]; (1) Absent [Supplementary data (Fig. 3.1)]	5	20	20
4	Head, pattern of spots, in dorsal view: (0) 1+1 dark, narrow and longitudinal, extending from the neck to the anteocular region [Supplementary data (Fig. 3.6)]; (1) Y-shaped over the clypeus [Supplementary data (Fig. 3.2)]; (2) Dark mottled [Supplementary data (Fig. 3.7)].	2	NI	NI
5	Neck, spots, in dorsal view: (0) Present [Supplementary data (Fig. 3.6)]; (1) Absent [Supplementary data (Fig. 3.8)]	2	50	0
6	Neck, 1+1 spots, in dorsal view: (0) Dark and longitudinal, forming narrow lines [Supplementary data (Fig. 3.6)]; (1) Punctuated lights [Supplementary data (Fig. 3.10)]; (2) Dark scored [Supplementary data (Fig. 3.7)]; (3) Uniformly yellow [Supplementary data (Fig. 3.9)]	3	NI	NI
7	Pronotum, collar, spots, in dorsal view: (0) Present [Supplementary data (Fig. 3.6)]; (1) Absent [Supplementary data (Fig. 3.1)]	3	33	33
8	Pronotum, collar, pattern of spots, in dorsal view: (0) 1+1 punctual dark spots [Supplementary data (Fig. 3.6)]; (1) Continuous light [Supplementary data (Fig. 3.9-10)]; (2) Dark mottled [Supplementary data (Fig. 3.7)].	2	NI	NI
9	Pronotum, posterior lobe, predominant colour, in dorsal view: (0) Brown clear [Supplementary data (Fig. 3.11)]; (1) Orange or yellowish [Supplementary data (Fig. 3.10)]; (2) Red [Supplementary data (Fig. 3.12)]; (3) Dark [Supplementary data (Fig. 3.13)]	8	37	37
10	Abdomen, connexivum, dorsal plates, spots: (0) Present [Supplementary data (Fig. 3.14)]; (1) Absent [Supplementary data (Fig. 3.15)].	1	NI	NI
11	Abdomen, connexivum, dorsal plates, stain pattern: (0) Dark in portions anterior, not covering the intersegmental sutures [Supplementary data (Fig. 3.14)]; (1) Dark and similar to musical notes, on the intersegmental sutures [Supplementary data (Fig. 3.16)]; (2) Dark and narrow over the intersegmental sutures [Supplementary data (Fig. 3.17)]; (3) Dark and wide over the intersegmental sutures [Supplementary data (Fig. 3.18)]; (4) Dark over the entire segment, except in the posterior part with a light stain [Supplementary data (Fig. 3.19)]; (5) Dark covering the entire segment, except the intersegmental sutures [Supplementary data (Fig. 3.20)]; (6) Dark and wide in the anterior portions, adjacent to the sutures intersegmental [Supplementary data (Fig. 3.21)]; (7) Dark sub-rectangular in the centre reaching the outer margin and other narrow margins over the intersegmental sutures [Supplementary data (Fig. 3.22)]; (8) Continuously dark and light inner and outer margins, respectively [Supplementary data (Fig. 3.23)].	16	50	50
12	Abdomen, colour pattern, in ventral view: (0) Complex [Supplementary data (Fig. 3.24)]; (1) Simple [Supplementary data (Fig. 3.25)].	2	50	50
13	Femurs, stains: (0) Present [Supplementary data (Fig. 3.26)]; (1) Absent [Supplementary data (Fig. 3.27)].	8	12	41
14	Femora, stain pattern: (0) Dark speckled [Supplementary data (Fig. 3.26)]; (1) Clear apical rings [Supplementary data (Fig. 3.28)]; (2) Clear submedian rings [Supplementary data (Fig. 3.29)]; (3) Dark apical rings [Supplementary data (Fig. 3.30)]; (4) Irregular browns [Supplementary data (Fig. 3.31)].	4	100	100
15	Tibiae, stains: (0) Present [Supplementary data (Fig. 3.26)]; (1) Absent [Supplementary data (Fig. 3.27-28)].	4	25	25
16	Tibiae, stain pattern: (0) Two dark rings, one basal and one submedian [Supplementary data (Fig. 3.26)]; (1) Dark apical rings [Supplementary data (Fig. 3.32)]; (2) Irregular browns [Supplementary data (Fig. 3.31)]; (3) Clear apical rings [Supplementary data (Fig. 3.31)]; (4) Clear basal rings [Supplementary data (Fig. 3.34)]	4	NI	NI
17	Head, space between the inner margins of the eyes, in dorsal view: (0) Smooth [Supplementary data (Fig. 3.6)]; (1) Weakly granular [Supplementary data (Fig. 3.1)]; (2) Weakly rough [Supplementary data (Fig. 3.35)]; (3) Strongly rough [Supplementary data (Fig. 3.36)].	7	42	60
18	Head, region between tubercle and eye, in lateral view: (0) Granular [Supplementary data (Fig. 3.4)]; (1) Rough [Supplementary data (Fig. 4A)]; (2) Smooth [Supplementary data (Fig. 3.5)].	7	28	58
19	Pronotum, posterior lobe, in dorsal view: (0) Granulose [Supplementary data (Fig. 3.11)]; (1) Rough [Supplementary data (Fig. 3.8,10,12,37-39)].	2	NI	NI
20	Thorax, propleura and metapleura, in lateral view: (0) Granulose [Supplementary data (Fig. 3.40)]; (1) Rough [Supplementary data (Fig. 3.41-44)].	2	50	75
21	Abdomen, sternites: (0) Smooth [Supplementary data (Fig. 3.24)]; (1) Striates [Supplementary data (Fig. 3.25)].	1	NI	NI
22	Head, shape, in dorsal view: (0) Subcylindrical [Supplementary data (Fig. 3.6)]; (1) Subsquare [Supplementary data (Fig. 3.2)]; (2) Subtriangular [Supplementary data (Fig. 3.7)].	2	100	100
23	Head, median longitudinal projection, in dorsal view: (0) Present [Supplementary data (Fig. 3.6)]; (1) Absent [Supplementary data (Fig. 3.1-3)].	1	NI	NI
24	Head, length in relation to width at eye level, in dorsal view: (0) Twice or as long as wide; (1) Less than twice as long as wide.	6	16	68
25	Head, length in relation to the length of the pronotum, in dorsal view: (0) As long as or, longer; (1) Shorter.	5	20	33
26	Anteocular region, length in relation to the postocular region, in dorsal view: (0) Less than three times longer; (1) Three to four times longer; (2) More than four to five times longer; (3) Six or more times longer.	11	27	65
27	Postocular region, shape of the margins, in dorsal view: (0) Strongly concave [Supplementary data (Fig. 3.6)]; (1) Substraight or weakly convex [Supplementary data (Fig. 3.1)]; (2) Strongly convex [Supplementary data (Fig. 3.7)].	5	40	25
28	Post-ocular region, callosities between the eye and ocelli, in lateral view: (0) Present [Supplementary data (Fig. 3.6)]; (1) Absent [Supplementary data (Fig. 3.1)].	1	100	100
29	Maxillary plate, length in relation to the apical margin of the postclypeus, in dorsal view: (0) Larger [Supplementary data (Fig. 3.6)]; (1) Smaller [Supplementary data (Fig. 3.35)].	2	50	83
30	Mandibular plate, pointed, in dorsal view: (0) Absent [Supplementary data (Fig. 3.6)]; (1) Discreet [Supplementary data (Fig. 3.36)]; (2) Well developed [Supplementary data (Fig. 3.35)].	6	33	20
31	Mandibular plate, length in relation to the maxillary plate, dorsal view: (0) Absent or obsolete; (1) At least 1/3 to 1/2; (2) Greater than or equal to 2/3.	5	40	50
32	Labium, third visible segment, length in relation to the second: (0) Shorter [Supplementary data (Fig. 3.4)]; (1) As long as or longer [Supplementary data (Fig. 3A)]	1	100	100

33	Labium, first visible segment, apex in relation to the antenniferous tubercle, in lateral view: (0) Reaching or exceeding the level of the anterior margin [Supplementary data (Fig. 3.4)]; (1) Not reaching the level of the anterior margin [Supplementary data (Fig. 3A)].	1	33	66
34	Labium, second visible segment, apex in relation to the eye, in lateral view: (0) Reaching or exceeding the level of the posterior margin [Supplementary data (Fig. 3.4,5)]; (1) Reaching the median level [Supplementary data (Fig. 3A)]; (2) Reaching the anterior margin.	4	50	33
35	Labium, third segment visible, shape of the apex, in ventral view: (0) Tapered [Supplementary data (Fig. 3.46)]; (1) Truncated [Supplementary data (Fig. 3.47)].	3	33	83
36	Eye, in relation to the lower margin of the ventral surface of the head, in lateral view: (0) Reaching or exceeding it [Supplementary data (Fig. 3.4)]; (1) Not reaching it.	6	16	58
37	Eye, length in relation to the total length of the head, dorsal view: (0) Less than or equal to 1/4; (1) Greater than 1/4.	3	33	0
38	Eye, width in relation to the space between the eyes, in dorsal view: (0) As wide as or larger; (1) Minor.	5	20	0
39	Approximation between the ocelli, dorsal view: (0) Proximal (Figures S3.6); (1) Distal [Supplementary data (Fig. 3.7)].	1	NI	NI
40	Antenniferous tubercles, location, in dorsal view: (0) Distal, close to the apex of the head [Supplementary data (Fig. 3.6)]; (1) Median, between the apex of the head and the eyes [Supplementary data (Fig. 3.1,3,9,36)]; (2) Proximal, close to the eyes [Supplementary data (Fig. 3.2,35)].	2	100	100
41	First antennal segment, position of the apex in relation to the apex of the clypeus, in dorsal view: (0) Not reaching it; (1) Reaching or exceeding it.	4	25	78
42	Third antennal segment, length in relation to the second: (0) Shorter; (1) As long or slightly longer.	2	50	50
43	Pronotum, submedian carina, extension: (0) Ending close to or reaching the distal margin of the posterior lobe [Supplementary data (Fig. 3.11)]; (1) Reaching only the submedian region of the posterior lobe [Supplementary data (Fig. 3.10-12)].	6	16	50
44	Pronotum, side carina: (0) Absent [Supplementary data (Fig. 3.11)]; (1) Present [Supplementary data (Fig. 3.45)].	2	50	0
45	Pronotum, shape of the side carina: (0) Smooth; (1) Wavy [Supplementary data (Fig. 3.45)].	1	NI	NI
46	Pronotum, anterolateral angles, length and width ratio: (0) Wider than long; (1) Longer than wide.	4	25	62
47	Pronotum, anterolateral angles, direction in relation to the longitudinal cephalic axis, in dorsal view: (0) Convergent [Supplementary data (Fig. 3.11)]; (1) Divergent [Supplementary data (Fig. 3.37-39)].	1	NI	NI
48	Pronotum, anterior lobe, latero-apical disc tubercles: (0) Absent or obsolete [Supplementary data (Fig. 3.11)]; (1) Developed [Supplementary data (Fig. 3.10,12,13)].	4	25	80
49	Pronotum, anterior lobe, basal disc tubercles: (0) Absent or obsolete [Supplementary data (Fig. 3.11)]; (1) Developed [Supplementary data (Fig. 3.13)].	7	14	45
50	Pronotum, anterior lobe, lateral tubercles: (0) Absent or obsolete [Supplementary data (Fig. 3.11)]; (1) Developed [Supplementary data (Fig. 3.38)].	5	20	76
51	Pronotum, posterior lobe, direction of the humeral angles, in lateral view: (0) Not elevated [Supplementary data (Fig. 3.40)]; (1) Reflected upwards [Supplementary data (Fig. 3.44)].	1	NI	NI
52	Pronotum, posterior lobe, humeral angles, elongated and tapered tubercles, in dorsal view: (0) Absent [Supplementary data (Fig. 3.11)]; (1) Presents [Supplementary data (Fig. 3.39)].	1	NI	NI
53	Scutellum, basal margin, triangular and tapered tubercles, in dorsal view: (0) Absent; (1) Presents.	1	NI	NI
54	Scutellum, lateral margin, anterior processes, in dorsal view: (0) Present; (1) Absent.	2	50	50
55	Scutellum, central depression: (0) Discreet [Supplementary data (Fig. 3.11)]; (1) Well developed [Supplementary data (Fig. 3.39)].	5	20	42
56	Scutellum, posterior process, length in relation to the main body of the scutellum, in dorsal view: (0) As long as or longer; (1) Shorter.	7	14	68
57	Scutellum, posterior process, shape, in dorsal view: (0) Conical and short [Supplementary data (Fig. 3.11)]; (1) Conical, elongated and tapered [Supplementary data (Fig. 3.39)]; (2) Cylindrical [Supplementary data (Fig. 3.16)].	11	18	59
58	Scutellum, posterior process, shape, in lateral view: (0) Straight, slightly elevated [Supplementary data (Fig. 3.40,41,44)]; (1) Straight, tapered and obliquely facing upward [Supplementary data (Fig. 3.43)]; (2) Concave [Supplementary data (Fig. 3.42)].	2	100	100
59	Scutellum, posterior process, apex shape, in lateral view: (0) Rounded [Supplementary data (Fig. 3.40,41,44)]; (1) Bifurcated [Supplementary data (Fig. 3.42)]; (2) Thin [Supplementary data (Fig. 3.43)].	2	NI	NI
60	Prosternum, prosternal groove, shape: (0) Long and narrow; (1) Short and wide.	2	50	85
61	Prosternum, prosternal groove, lateral margins: (0) Continuously narrowing from base to apex; (1) Straight, parallel, narrowing in the apical third.	3	33	66
62	Hemelytron, length, in dorsal view: (0) Reaching or slightly exceeding the apex of the seventh urotergite [Supplementary data (Fig. 3.14)]; (1) Reaching the anterior edge of the sixth urotergite.	1	50	0
63	Hemelytron, small branch connecting the basal portion R + M to S: (0) Absent [Supplementary data (Fig. 3.14)]; (1) Present [Supplementary data (Fig. 3.16)].	1	100	100
64	Abdomen, width in relation to the total length of the body: (0) Less than half [Supplementary data (Fig. 3.14)]; (1) Half or more.	1	NI	NI
65	Abdomen, suture of the urosternite plate in relation to the dorsal plate of the connective, ventral view: (0) Proximal [Supplementary data (Fig. 3.24)]; (1) Distal; (2) Pleated membrane longitudinally conspicuous connecting the plates [Supplementary data (Fig. 3.25)].	2	100	100
66	Abdomen, connexivum, ventral plates: (0) Visible (Figure x); (1) Not visible [Supplementary data (Fig. 3.25)].	1	NI	NI
67	Abdomen, connexivum, pleated membrane connecting the dorsal plates to the sternites: (0) Absent; (1) Present [Supplementary data (Fig. 3.48)].	1	NI	NI
68	Connexivum, ventral plates: (0) Noticeable, similar in width to that shown dorsally; (1) Not noticeable, conspicuous longitudinally [Supplementary data (Fig. 3.48)].	1	NI	NI

L: length; CI: consistency index; RI: retention index; NI: not informative.

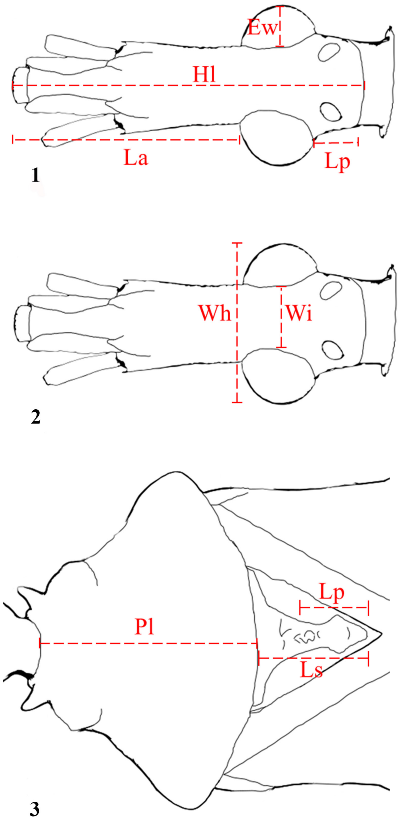

Fig. 1: (1-2) Head, dorsal view. (3) Pronotum and Scutellum, dorsal view. Hl: head length, Ew: eye width, La: length of the anteocular region, Lp: length of the postocular region, Wh: maximum width of the head between the eyes, Wi: width of the interocular space, Pl: pronotum length, Ls: length of the scutellum, Lp: length of the scutellum process.

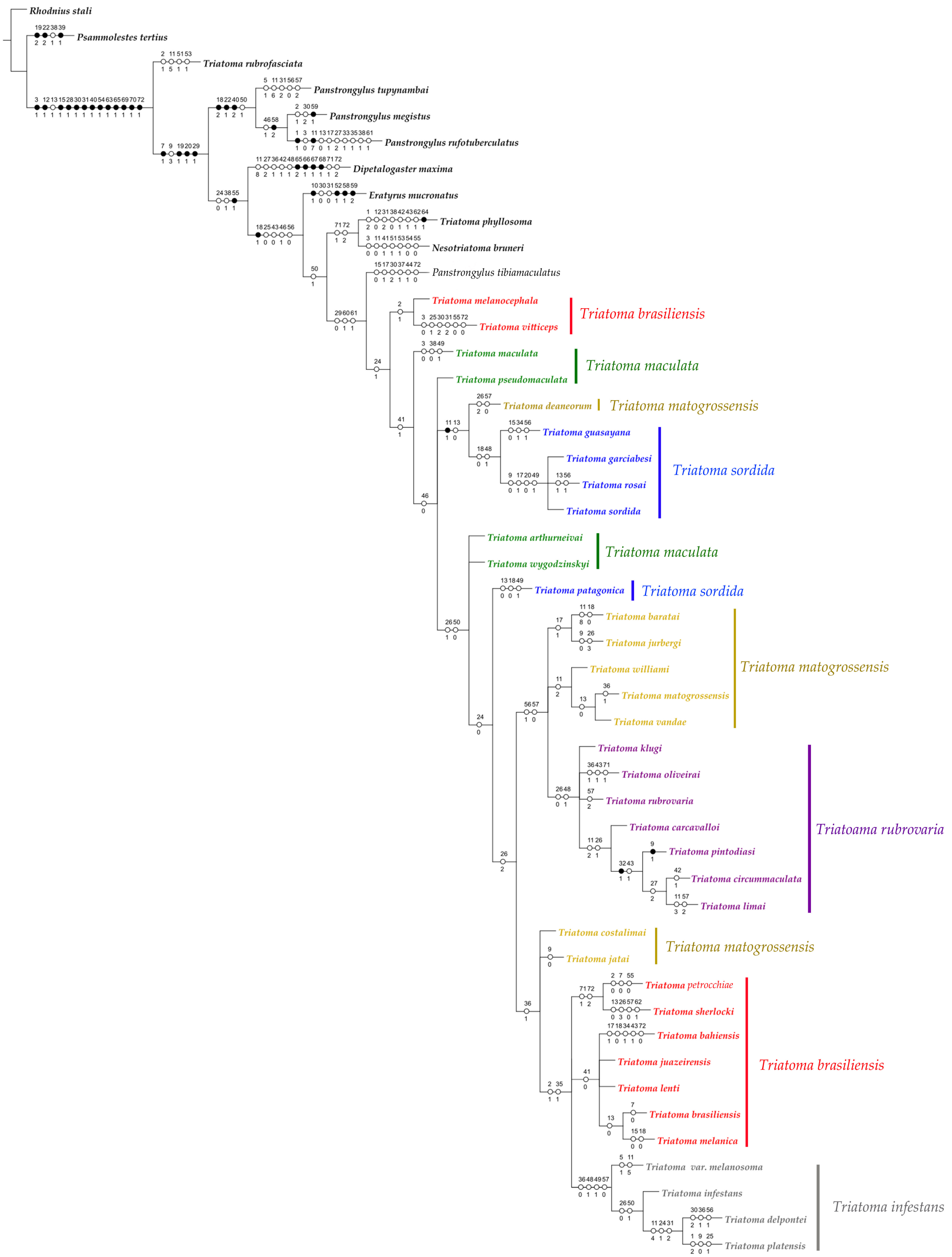

Fig. 2: strict consensus of the three most parsimonious trees that resulted from the analysis under implicit weights with K = 3 (Fit = 2.18956, L = 250, CI = 0.41, RI = 0.59), and their characters and states. The classification follows.[7,49] Terminal taxa in color: black = outgroup; red = *Triatoma brasiliensis*; green = *Triatoma maculata*; yellow = *Triatoma matogrossensis*; purple = *Triatoma rubrovaria*; and grey = *Triatoma infestans*.

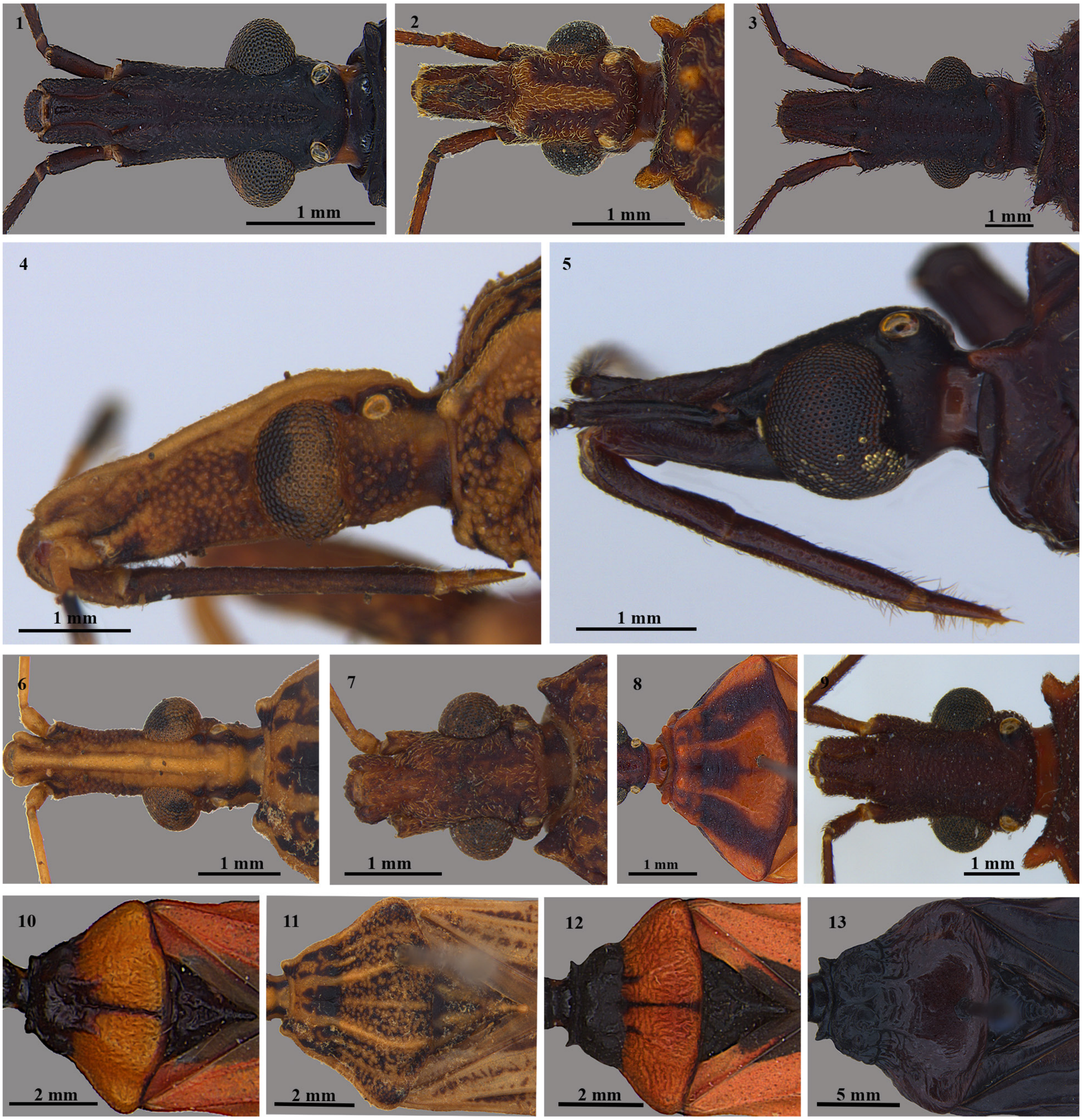

Fig. 3: (1-13) Head, dorsal view, 1. *Triatoma baratai*, 2. *Panstrongylus rubrotuberculatus*, 3. *Triatoma phyllosoma*. Head, lateral view, 4. *Rhodnius stali*, 5. *Panstrongylus megistus*. Head, dorsal view, 6. *R. stali*, 7. *Psammolestes tertius*. Pronotum dorsal view, 8. *Panstrongylus tupynambai*. Head, dorsal view, 9. *Triatoma rubrofasciata*. Pronotum and scutellum, dorsal view, 10. *Triatoma pintodiasi*. 11. *R. stali*, 12. *Triatoma carcavalloi*, 13. *Dipetalogaster maxima*.

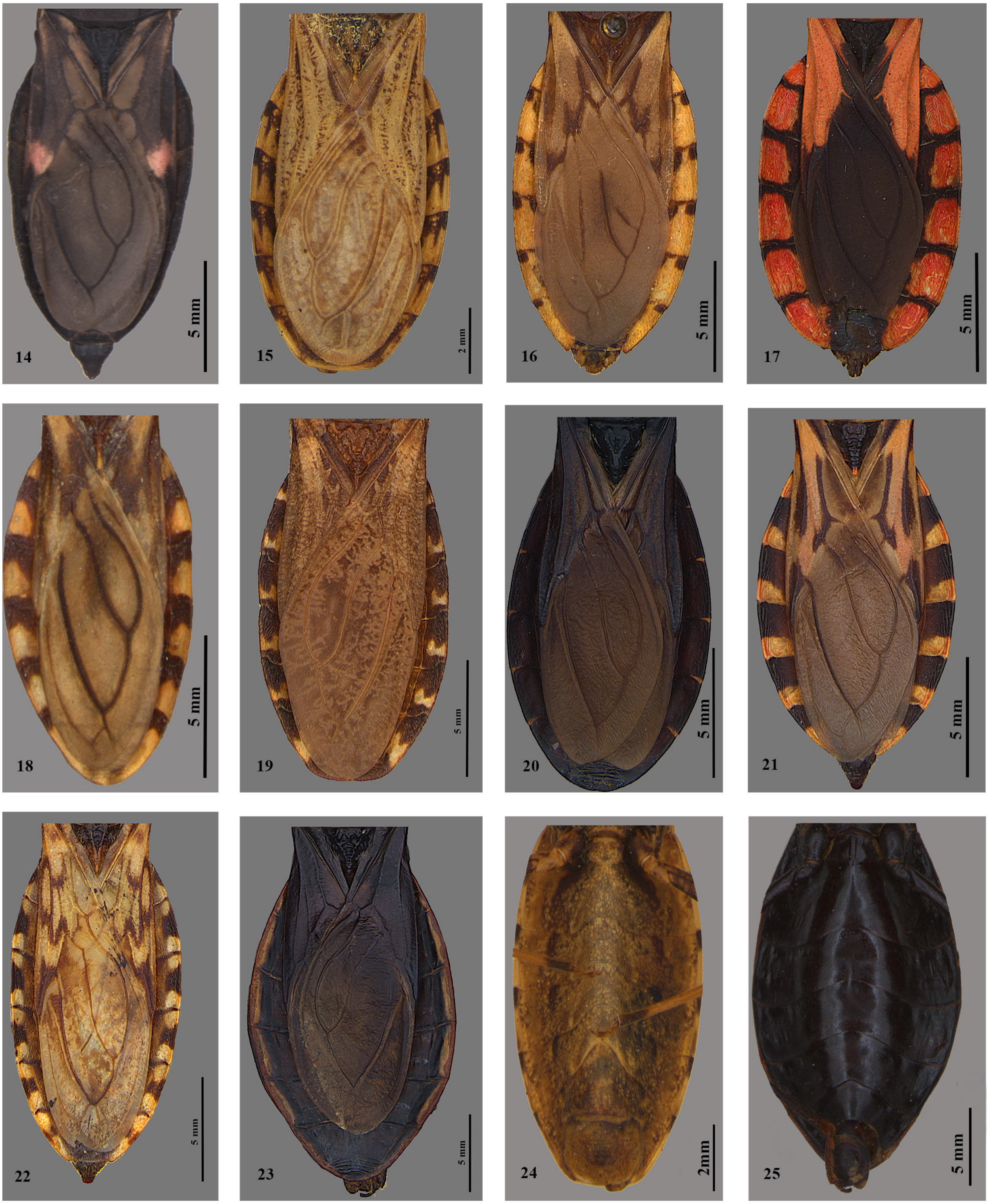

Fig. 3: (14-25) Posterior portion of body, including connexivum, in dorsal view: 14. *Eratyrus mucronatus*, 15. *Rhodnius stali*, 16. *Triatoma sordida*, 17. *Triatoma carcavalloi*, 18. *Triatoma brasiliensis*, 19. *Triatoma delpontei*, 20. *Triatoma infestans* var. *melanosoma*, 21. *Panstrongylus tibiamaculatus*, 22. *Panstrongylus rufotuberculatus*, 23. *Dipetalogaster maxima,* in ventral view, 24. *R. stali*, 25. *D. maxima.*

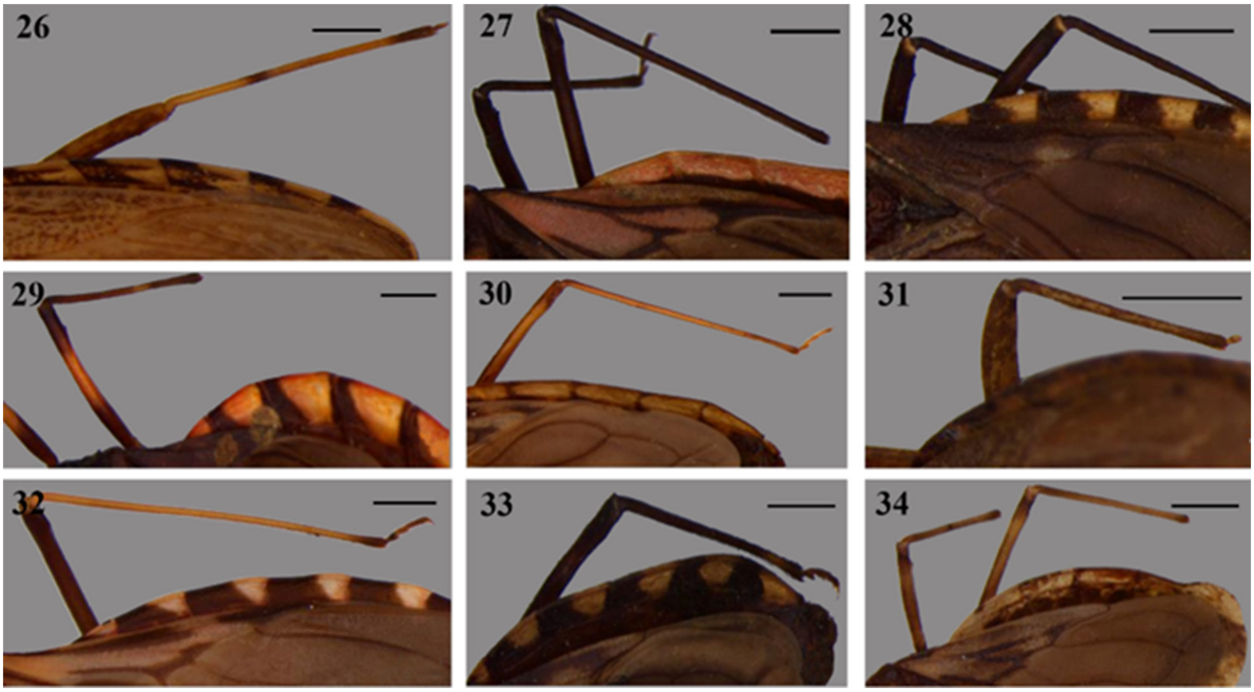

Fig. 3: (26-37) Hind leg, dorsal view: 26. *Rhodnius stali*, 27. *Triatoma baratai*, 28. *Triatoma patagonica*, 29. *Triatoma sherlocki*, 30. *Triatoma matogrossensis*, 31. *Psamolestes tertius*, 32. *Panstrongylus tibiamaculatus*, 33. *Triatoma melanica*, 34. *Triatoma guasayana*. Scale: 2 mm.

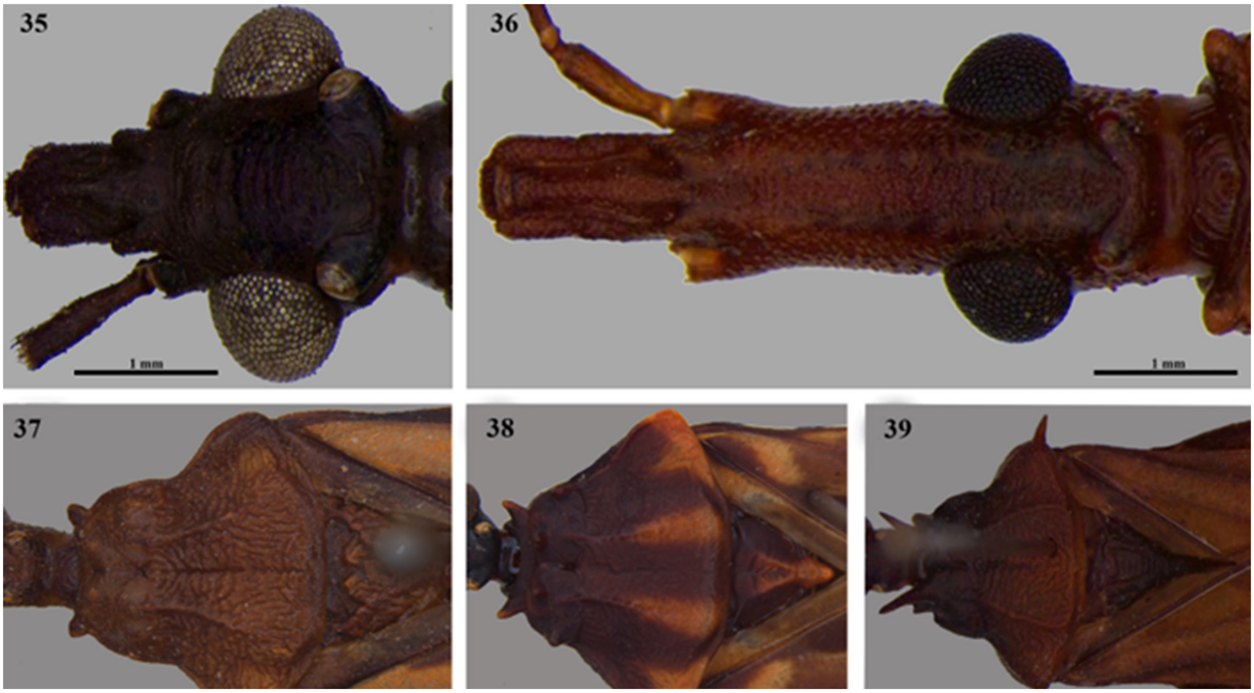

Fig. 3: (35-39) Head, 35. *Panstrongylus megistus*, 36. *Triatoma matogrossensis*, dorsal view. Pronotum, 37. *T. matogrossensis*, 38. *P. megistus*, 39. *Eratyrus mucronatus*, dorsal view.

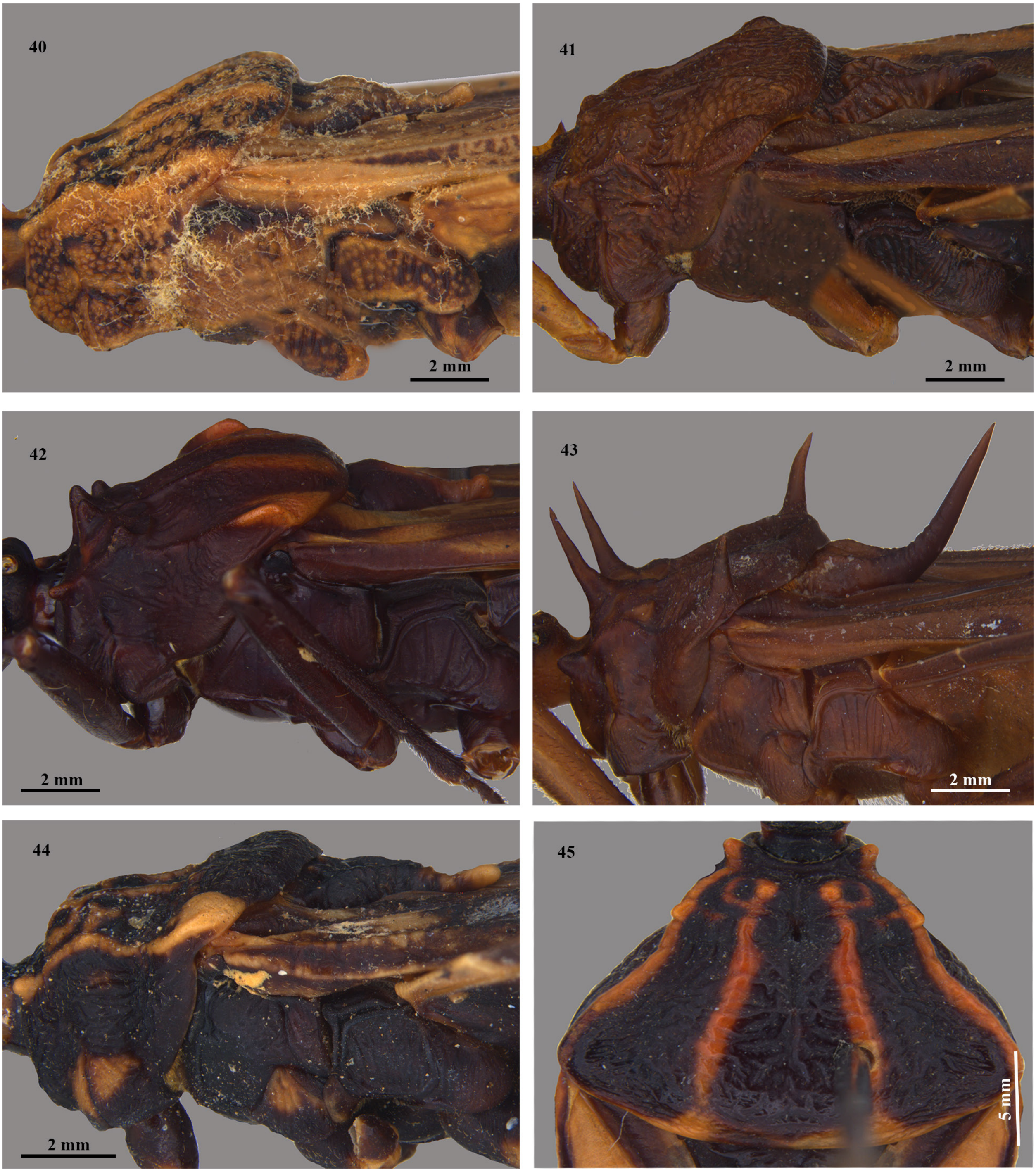

Fig. 3: (40-45) Pronotum, 40. *Rhodnius stali*, 41. *Triatoma matogrossensis*, 42. *Panstrongylus megistus*, 43. *Eratyrus mucronatus*, 44. *Nesotriatoma bruneri,* lateral view. 45. Pronotum, *Panstrongylus tibiamaculatus*, dorsal view.

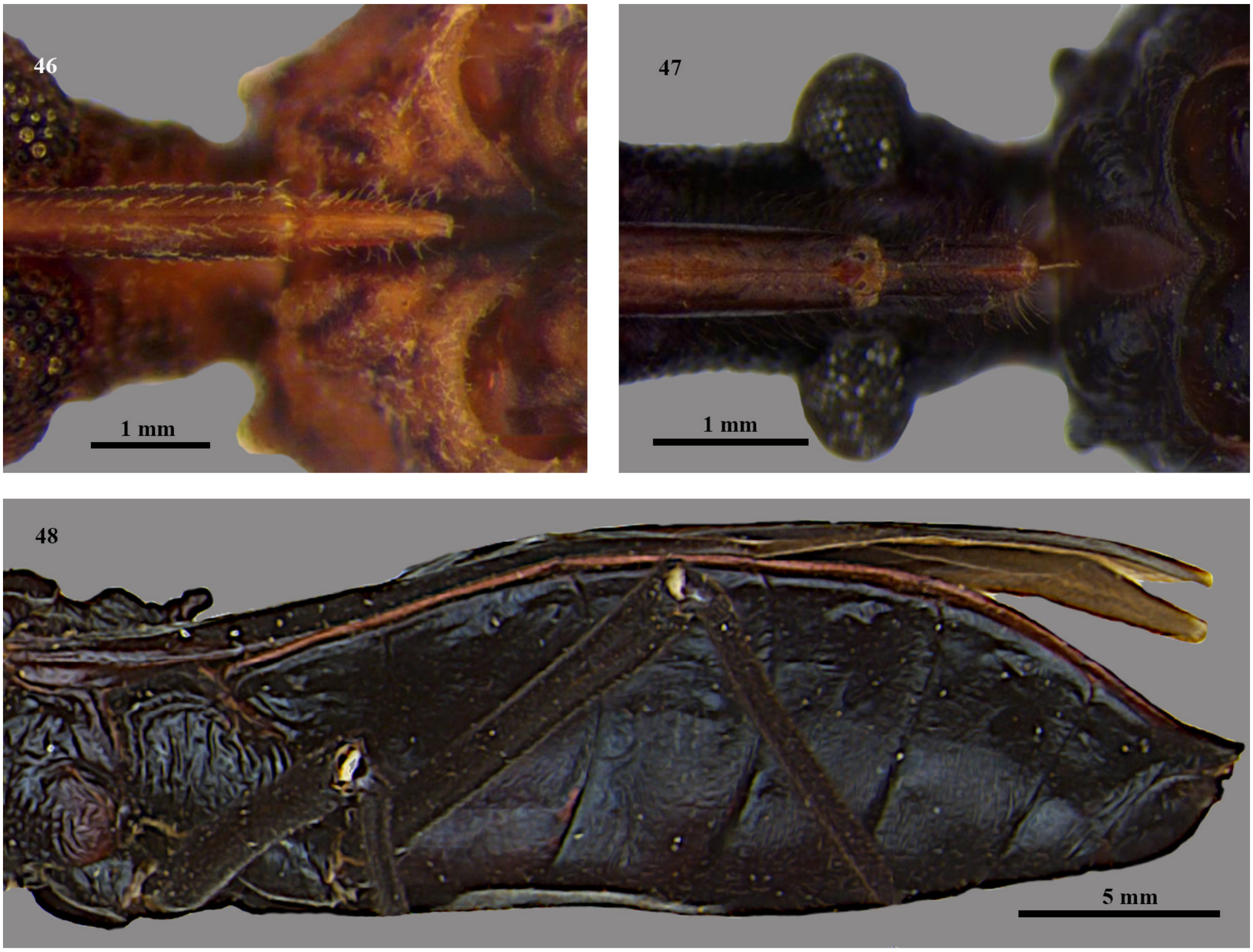

Fig. 3: (46-48) Third visible labial segment, 44. *Rhodnius stali*, 45. *Triatoma brasiliensis*, ventral view. 48. Abdomen, *Dipetalogaster maxima*, lateral view.
