# Supplementary material for: Cladistics of South American Triatoma (Hemiptera: Reduviidae): a morphological overview of T. infestans complex
Source: Mem Inst Oswaldo Cruz. 2026 Jul 20;121(Suppl 1):e250293. doi: 10.1590/0074-02760250293 (PMC13395083; doi:10.1590/0074-02760250293)
Supplement: Peer review history

# Reviewer report: FIRST REVIEW ROUND - REVIEWERS COMMENTS

## REVIEWER #1

Dear authors,

The manuscript presents an important study on the *Triatoma infestans* complex. Therefore, several aspects of this work deserve attention before publication.

There are already robust doctoral theses on the web that have not been published due to a lack of support in the morphological cladistics of these species.

Many groups, when working with cladistics, use representatives from more distant groups to better root the trees; I believe you are aware of this. Aspects such as the lack of supplementary table 5 with morphological characters are difficult to evaluate. I have made other serious suggestions in the attached file that should be considered. Some references are already outdated (as in the second paragraph of the introduction). We need to know the reason for the choice of species as "outgroup".

I believe that this manuscript, in its current form, should not be published in Memórias.

I await news.

All the best.

## REVIEWER #2

This manuscript provides the most comprehensive morphological cladistic analysis to date of South American *Triatoma*, particularly the *T. infestans* complex. The study is methodologically sound and based on extensive examination of type and reference specimens. The use of both equal and implied weighting, coupled with bootstrap and Bremer support, is appropriate and strengthens the morphological framework presented.

Nevertheless, the paper would benefit from a more critical discussion of the continued use of traditional subcomplexes. The results clearly demonstrate that several of these assemblages are not monophyletic, consistent with extensive molecular and cytogenetic evidence (e.g., for *T. guasayana*, *T. patagonica*, *T. maculata*). Maintaining these outdated categories risks obscuring, rather than clarifying, the true evolutionary relationships within *Triatoma*. I strongly encourage the authors to address whether the subcomplex framework remains conceptually valid or should be replaced by clades supported by integrative data.

Additionally, Figures 1 and 2 visually group taxa under color codes corresponding to these non-monophyletic subcomplexes. This graphical choice may mislead readers by implying monophyly where it is not supported. I recommend revising the figures so that colors or labels only represent recovered monophyletic clades, or alternatively, indicate non-monophyletic assemblages with neutral tones or dashed outlines.

Overall, this study is a valuable contribution to Triatominae systematics, and its impact would be enhanced by aligning the interpretation and visual presentation with the phylogenetic results and with modern integrative frameworks.

## AUTHORS' RESPONSE TO THE REVIEWERS

Dear Dr. Adeilton Brandão,

Please find below a detailed document containing our responses to the reviewers' comments.

We sincerely appreciate the time and valuable suggestions provided by the reviewers, which have significantly contributed to improving the quality and clarity of our work. All comments were carefully addressed in the revised manuscript, and the corresponding changes are clearly indicated.

Thank you for your consideration.

## Reviewer 1

Dear authors,

The manuscript presents an important study on the *Triatoma infestans* complex. Therefore, several aspects of this work deserve attention before publication.

There are already robust doctoral theses on the web that have not been published due to a lack of support in the morphological cladistics of these species.

Many groups, when working with cladistics, use representatives from more distant groups to better root the trees; I believe you are aware of this. Aspects such as the lack of supplementary table 5 with morphological characters are difficult to evaluate. I have made other serious suggestions in the attached file that should be considered. Some references are already outdated (as in the second paragraph of the introduction). We need to know the reason for the choice of species as "outgroup".

I believe that this manuscript, in its current form, should not be published in Memórias.

I await news.

All the best.

**A:** Dear Reviewer,

We appreciate your careful reading and detailed considerations regarding our manuscript, as well as your recognition of the relevance of the study on the *Triatoma infestans* complex. Your observations were fundamental to the improvement of the manuscript.

Regarding the observation about the existence of unpublished theses due to the fragility of morphological cladistic support, we emphasize that the present study was conducted with rigorous criteria for taxon selection, encompassing most species of the complex and a greater number of morphological characters compared to previous studies. The character set was thoroughly reviewed and subjected to parsimony analyses with different search strategies, aiming for greater robustness and support for the recovered clades.

The choice of the outgroup is justified in the manuscript, considering that our study seeks to observe the phylogenetic relationships of some *Triatoma* species, and excessively distant taxa can compromise character polarization, correct rooting of the tree, and increase homoplasies.

Regarding the absence of Supplementary Table 5, we acknowledge the error in the submission. The table, containing the complete and detailed list of morphological characters and their states, has now been submitted. The other suggestions were included. The modifications made were substantial, increasing the clarity of the text and the analyses.

**Reviewer:** Why not use the paper that described the nineteenth genera?

**A:** The suggestion was incorporated. In addition, the species number was updated, as well as the citation of the most recently described species, *Triatoma chiarii*.

**Reviewer:** The references are very outdated; we have new groups being proposed now.

**A:** Since Schofield & Galvão (2009), *Triatoma* has traditionally been divided into three major groups: *rubrofasciata*, *dispar*, and *infestans*. To date, this general classification has been maintained. However, several modifications have been proposed within these groups, including the establishment of new subcomplexes, the suppression or redefinition of others, as well as changes in their composition. As discussed further below in the text.

Additionally, the number of existing species in *Triatoma* was modified from 75 to 76 species due to the description of *Triatoma chiarii*.

**Reviewer:** I was only able to view 47 references.

**A:** Typo. Corrected. Instead of 76, it was changed to 7.

**Reviewer:** Add reference Schofield and Galvão 2009?

**A:** Done.

**Reviewer:** (7-9)

**A:** Done. With reference to the description of *Triatoma chiarii*. Thus, it became (8-10).

**Reviewer:** In fact, the authors need to better explain why they chose these species, since only triatomines were selected.

**A:** The text has been modified for better understanding.

**Reviewer:** When we talk about cladistics, your selected species are all representative of Triatominae, and we needed representatives of Reduviidae, as in the works you referenced.

**A:** The use of excessively distant taxa as an outgroup is not recommended, since large phylogenetic distances can compromise the proper polarization of characters and the correct rooting of the tree. Very distant outgroups tend to introduce homoplasies, long-branch attraction effects, and ambiguities in the interpretation of character states, reducing the reliability of phylogenetic inferences. Therefore, the outgroup should be phylogenetically close enough to the ingroup to share homologous characters, but clearly external to it, ensuring more robust and consistent analyses. In this study, the ingroup consisted exclusively of South American species of *Triatoma*; for this reason, species that were not so distant, as well as the Old-World species *Triatoma rubrofasciata* and representatives of other genera from the tribes Triatomini and Rhodniini, were used as outgroups. Therefore, it is plausible to restrict the ingroup to triatomines.

**Reviewer:** Common museum abbreviation: MfN.

**A:** Done.

**Reviewer:** Why weren't the specimens from the outgroup listed here?

**A:** The outgroup is listed in the supplementary material.

**Reviewer:** When using specimens that are not from the type series, a bias is created with the phenotypic characteristics.

**A:** This is a typographical error. The type specimen of *Triatoma brasiliensis* has been analyzed and is deposited in the Coleção Entomológica de Instituto Oswaldo Cruz (CEIOC), as indicated in the supplementary material. The text has been corrected.

**Reviewer:** Several species were analyzed through photography; how can we be sure of the information?

**A:** The use of photographs as a data source was carried out carefully and did not replace, when available, the direct analysis of specimens. The images used correspond to high-resolution photographs from the study by Rodrigues et al (2020). List of the type specimens of Triatominae (Hemiptera: Heteroptera: Reduviidae) in the Hemimetabola Collection of the Museum für Naturkunde, Berlin. Zootaxa. 2020; 4809 (2): 329-348. These images allowed for the reliable observation of diagnostic morphological characters widely used in the taxonomy of Triatominae. Whenever possible, the information obtained through photographs was compared with original descriptions, taxonomic revisions, and directly examined material, ensuring the consistency and reliability of the data used.

**Reviewer:** I believe it is necessary to compile a list of the localities of each specimen used that are not "types". Especially for these cosmopolitan species.

**A:** The list can be found in the supplementary material.

**Reviewer:** We don't know how many specimens or the sex of each species was used.

**A:** The list is in the supplementary material. However, the quantity has been included in the text.

**Reviewer:** Using TNT nowadays is obsolete. I believe that for the dataset, 5000 replications is not an exhaustive analysis.

**A:** We recognize that there are currently several approaches and software widely used in phylogenetic inference. However, we opted for the use of TNT due to its recognized suitability for the analysis of morphological data and its proven efficiency in parsimony-based inferences. To ensure the robustness of the results, we performed 5,000 replications, employing equal and implicit weighting, as well as advanced heuristic search algorithms (e.g., New Technology Search, including sectoral searches, ratchet, and tree drifting). Additionally, independent searches were conducted, all converging to consistent topologies, indicating adequate exploration.

Recently, Goloboff & Morales (2023) presented an updated version of TNT, demonstrating its effectiveness in inferring trees with groups of different quantities of taxa and in extensive datasets, in addition to highlighting the program's efficiency in parsimony analyses, especially when combined with advanced heuristic strategies applied to morphological data. Recent studies corroborate the continued use of TNT as a robust tool for parsimony inference in insect morphological matrices. For example, analyses with beetles of the genus *Tiaronthophagus* employed TNT for the simultaneous evaluation of discrete and continuous characters, using multiple heuristic search methods; Molina & Gil-Azevedo (2021) proposed the phylogeny of neotropical and afrotropical *Simulium* based on parsimonious analyses; Gutérrez-Tejo et al. (2024) used TNT v1.6 with new technology search strategies and bootstrap support to infer phylogenetic relationships among *Tetraopes* species; and Li et al. (2025) applied TNT in analyses of Neuroptera (Mantispoidea), combining traditional and advanced searches and reporting node support by bootstrap and Bremer indices. Taken together, these examples demonstrate that TNT remains widely used in recent literature, particularly in phylogenetic studies of insects based on morphological characters or integrated datasets.

References

Roggero, A.; Moretto, P.; Barbero, E.; Palestrini, C. The Phylogenetic Relationships of *Tiaronthophagus* n.gen. (Coleoptera, Scarabaeidae, Onthophagini) Evaluated by Phenotypic Characters. Insects 2019, 10, 64. https://doi.org/10.3390/insects10030064

Molina Ó S; Gil-Azevedo, Leonardo H. *Simulium* (*Trichodagmia*) (Diptera, Simuliidae) phylogeny revisited: the Neotropical and Afrotropical connection. European Journal of Taxonomy, v. 773, p. 80-119, 2021. https://doi.org/10.5852/ejt.2021.773.1517

Gutiérrez-Trejo, N., Van Dam, M.H., Lam, A.W. et al. Phylogenomics of *Tetraopes* longhorn beetles unravels their evolutionary history and biogeographic origins. Sci Rep 14, 7285 (2024). https://doi.org/10.1038/s41598-024-57827-z

Goloboff, P. A., & Morales, J. C. (2023). TNT version 1.6, with a graphical interface for MacOS and Linux, including parallelization. Cladistics, 39(2), 144–153. https://doi.org/10.1111/cla.12524

Hongyu Li, De Zhuo, Bo Wang, Hiroshi Nakamine, Shûhei Yamamoto, Weiwei Zhang, James E Jepson, Michael Ohl, Ulrike Aspöck, Horst Aspöck, Thet Tin Nyunt, Horst Aspöck, Thet Tin Nyunt, Michael S Engel, Michael J Benton, Philip Donoghue, Xingyue Liu, A Double-Edged Sword: Evolutionary Novelty Along Deep-Time Diversity Oscillation in an Iconic Group of Predatory Insects (Neuroptera: Mantispoidea), Systematic Biology, Volume 74, Issue 3, May 2025, Pages 395–420, https://doi.org/10.1093/sysbio/syae068

**Reviewer:** Here we can see an inconsistency that the limited data provides; *Triatoma rubrofasciata* could not be *Triatoma*, according to your inference.

**A:** Considering that the objective of the analysis was exclusively to investigate the phylogenetic relationships between the species of the *Triatoma infestans* complex and its respective subcomplexes, it was not intended to evaluate whether *Triatoma rubrofasciata* belongs to the genus *Triatoma* or not. A proper definition of the taxonomic position of *T. rubrofasciata* would require a more comprehensive phylogenetic analysis, including all species of the genus, with the incorporation of taxa from North America, the Old World and *Linshcosteus*, to which this species is most closely related (according to Schofield & Galvão, 2009; Justi et al., 2014, 2016; Kieran et al., 2021), in addition to species from the Andes, other regions of South America, and the Spinolai complex.

Reference:

Schofield CJ, Galvão C. Classification, evolution, and species groups within the Triatominae. Acta Tropica. 2009; 110: 88-100.

Justi SA, Russo CA, Mallet JR, Obara MT, Galvão C. Molecular phylogeny of Triatomini (Hemiptera: Reduviidae: Triatominae). Parasites & Vectors. 2014; 7: 137-149.

Justi SA, Galvão C, Schrago CG. Geological changes of the Americas and their influence on the diversification of the Neotropical kissing bugs (Hemiptera: Reduviidae: Triatominae). Plos Neglected Tropical Diseases. 2016; 10 (4): 1-22.

Kieran TJ, Gordon ERL, Zaldívar-Riverón A, Ibara-Cerdeña CN, Glenn TC, Weirauch C. Ultraconserved elements reconstruct the evolution of Chagas disease vectoring kissing bugs (Reduviidae: Triatominae). Systematic Entomology, 2021; 1-16.

**Reviewer:** We can see some inconsistencies here. This needs to be explained in the discussion since it contradicts more recent work.

**A:** The suggestions were included in the text.

## Reviewer 2

This manuscript provides the most comprehensive morphological cladistic analysis to date of South American *Triatoma*, particularly the *T. infestans* complex. The study is methodologically sound and based on extensive examination of type and reference specimens. The use of both equal and implied weighting, coupled with bootstrap and Bremer support, is appropriate and strengthens the morphological framework presented.

Nevertheless, the paper would benefit from a more critical discussion of the continued use of traditional subcomplexes. The results clearly demonstrate that several of these assemblages are not monophyletic, consistent with extensive molecular and cytogenetic evidence (e.g., for *T. guasayana*, *T. patagonica*, *T. maculata*). Maintaining these outdated categories risks obscuring, rather than clarifying, the true evolutionary relationships within *Triatoma*. I strongly encourage the authors to address whether the subcomplex framework remains conceptually valid or should be replaced by clades supported by integrative data.

Additionally, Figures 1 and 2 visually group taxa under color codes corresponding to these non-monophyletic subcomplexes. This graphical choice may mislead readers by implying monophyly where it is not supported. I recommend revising the figures so that colors or labels only represent recovered monophyletic clades, or alternatively, indicate non-monophyletic assemblages with neutral tones or dashed outlines.

Overall, this study is a valuable contribution to Triatominae systematics, and its impact would be enhanced by aligning the interpretation and visual presentation with the phylogenetic results and with modern integrative frameworks.

**A:** We appreciate the constructive comments, as well as the positive assessment regarding the comprehensiveness and analytical rigor of the manuscript. The discussion was implemented to critically address these points, as well as the other suggestions.

The combined use of equal and implicit weighting, together with supporting metrics, is especially appreciated, as it reinforces the consistency of the presented morphological structure reinforces the consistency of the presented morphological structure. The results obtained show that several subcomplexes recognized within *Triatoma* are not monophyletic groups, consistent with independent evidence from molecular and cytogenetic data. This issue remains central and there is no consensus.

We opted to maintain the use of colors in Figures 1 and 2, since this scheme follows the pattern employed in previous studies on the subcomplexes and species of the *Triatoma infestans* complex and related groups, facilitating the comparison and discussion of the results in the context of previous literature. We emphasize that, throughout the manuscript, we explicitly stated that these chromatic groupings correspond to historically proposed and admittedly non-monophyletic subcomplexes, being used only as a visual and comparative resource. Even so, we revised the legends and the text to make this caveat even clearer, minimizing any possibility of misinterpretation.

Below are other issues highlighted by the reviewers.

**Reviewer:** Why not use the paper that described the nineteenth genera?

**A:** The suggestion was incorporated. In addition, the species number was updated, as well as the citation of the most recently described species, *Triatoma chiarii*.

**Reviewer:** The references are very outdated; we have new groups being proposed now.

**A:** Since Schofield & Galvão (2009), *Triatoma* has traditionally been divided into three major groups: *rubrofasciata*, *dispar*, and *infestans*. To date, this general classification has been maintained. However, several modifications have been proposed within these groups, including the establishment of new subcomplexes, the suppression or redefinition of others, as well as changes in their composition. As discussed further below in the text.

Additionally, the number of existing species in *Triatoma* was modified from 75 to 76 species due to the description of *Triatoma chiarii*.

**Reviewer:** I was only able to view 47 references.

**A:** Typo. Corrected. Instead of 76, it was changed to 7.

**Reviewer:** Add reference Schofield and Galvão 2009?

**A:** Done.

**Reviewer:** (7-9)

**A:** Done. With reference to the description of *Triatoma chiarii*. Thus, it became (8-10).

**Reviewer:** In fact, the authors need to better explain why they chose these species, since only triatomines were selected.

**A:** The text has been modified for better understanding.

**Reviewer:** When we talk about cladistics, your selected species are all representative of Triatominae, and we needed representatives of Reduviidae, as in the works you referenced.

**A:** The use of excessively distant taxa as an outgroup is not recommended, since large phylogenetic distances can compromise the proper polarization of characters and the correct rooting of the tree. Very distant outgroups tend to introduce homoplasies, long-branch attraction effects, and ambiguities in the interpretation of character states, reducing the reliability of phylogenetic inferences. Therefore, the outgroup should be phylogenetically close enough to the ingroup to share homologous characters, but clearly external to it, ensuring more robust and consistent analyses. In this study, the ingroup consisted exclusively of South American species of *Triatoma*; for this reason, species that were not so distant, as well as the Old-World species *Triatoma rubrofasciata* and representatives of other genera from the tribes Triatomini and Rhodniini, were used as outgroups. Therefore, it is plausible to restrict the ingroup to triatomines.

**Reviewer:** Common museum abbreviation: MfN.

**A:** Done.

**Reviewer:** Why weren't the specimens from the outgroup listed here?

**A:** The outgroup is listed in the supplementary material.

**Reviewer:** When using specimens that are not from the type series, a bias is created with the phenotypic characteristics.

**A:** This is a typographical error. The type specimen of *Triatoma brasiliensis* has been analyzed and is deposited in the Coleção Entomológica de Instituto Oswaldo Cruz (CEIOC), as indicated in the supplementary material. The text has been corrected.

**Reviewer:** Several species were analyzed through photography; how can we be sure of the information?

**A:** The use of photographs as a data source was carried out carefully and did not replace, when available, the direct analysis of specimens. The images used correspond to high-resolution photographs from the study by Rodrigues et al (2020). List of the type specimens of Triatominae (Hemiptera: Heteroptera: Reduviidae) in the Hemimetabola Collection of the Museum für Naturkunde, Berlin. Zootaxa. 2020; 4809 (2): 329-348. These images allowed for the reliable observation of diagnostic morphological characters widely used in the taxonomy of Triatominae. Whenever possible, the information obtained through photographs was compared with original descriptions, taxonomic revisions, and directly examined material, ensuring the consistency and reliability of the data used.

**Reviewer:** I believe it is necessary to compile a list of the localities of each specimen used that are not "types". Especially for these cosmopolitan species.

**A:** The list can be found in the supplementary material.

**Reviewer:** We don't know how many specimens or the sex of each species was used.

**A:** The list is in the supplementary material. However, the quantity has been included in the text.

**Reviewer:** Using TNT nowadays is obsolete. I believe that for the dataset, 5000 replications is not an exhaustive analysis.

**A:** We recognize that there are currently several approaches and software widely used in phylogenetic inference. However, we opted for the use of TNT due to its recognized suitability for the analysis of morphological data and its proven efficiency in parsimony-based inferences. To ensure the robustness of the results, we performed 5,000 replications, employing equal and implicit weighting, as well as advanced heuristic search algorithms (e.g., New Technology Search, including sectoral searches, ratchet, and tree drifting). Additionally, independent searches were conducted, all converging to consistent topologies, indicating adequate exploration.

Recently, Goloboff & Morales (2023) presented an updated version of TNT, demonstrating its effectiveness in inferring trees with groups of different quantities of taxa and in extensive datasets, in addition to highlighting the program's efficiency in parsimony analyses, especially when combined with advanced heuristic strategies applied to morphological data. Recent studies corroborate the continued use of TNT as a robust tool for parsimony inference in insect morphological matrices. For example, analyses with beetles of the genus *Tiaronthophagus* employed TNT for the simultaneous evaluation of discrete and continuous characters, using multiple heuristic search methods; Molina & Gil-Azevedo (2021) proposed the phylogeny of neotropical and afrotropical *Simulium* based on parsimonious analyses; Gutérrez-Tejo et al. (2024) used TNT v1.6 with new technology search strategies and bootstrap support to infer phylogenetic relationships among *Tetraopes* species; and Li et al. (2025) applied TNT in analyses of Neuroptera (Mantispoidea), combining traditional and advanced searches and reporting node support by bootstrap and Bremer indices. Taken together, these examples demonstrate that TNT remains widely used in recent literature, particularly in phylogenetic studies of insects based on morphological characters or integrated datasets.

References

Roggero, A.; Moretto, P.; Barbero, E.; Palestrini, C. The Phylogenetic Relationships of *Tiaronthophagus* n.gen. (Coleoptera, Scarabaeidae, Onthophagini) Evaluated by Phenotypic Characters. Insects 2019, 10, 64. https://doi.org/10.3390/insects10030064

Molina Ó S; Gil-Azevedo, Leonardo H. *Simulium* (*Trichodagmia*) (Diptera, Simuliidae) phylogeny revisited: the Neotropical and Afrotropical connection. European Journal of Taxonomy, v. 773, p. 80-119, 2021. https://doi.org/10.5852/ejt.2021.773.1517

Gutiérrez-Trejo, N., Van Dam, M.H., Lam, A.W. et al. Phylogenomics of *Tetraopes* longhorn beetles unravels their evolutionary history and biogeographic origins. Sci Rep 14, 7285 (2024). https://doi.org/10.1038/s41598-024-57827-z

Goloboff, P. A., & Morales, J. C. (2023). TNT version 1.6, with a graphical interface for MacOS and Linux, including parallelization. Cladistics, 39(2), 144–153. https://doi.org/10.1111/cla.12524

Hongyu Li, De Zhuo, Bo Wang, Hiroshi Nakamine, Shûhei Yamamoto, Weiwei Zhang, James E Jepson, Michael Ohl, Ulrike Aspöck, Horst Aspöck, Thet Tin Nyunt, Horst Aspöck, Thet Tin Nyunt, Michael S Engel, Michael J Benton, Philip Donoghue, Xingyue Liu, A Double-Edged Sword: Evolutionary Novelty Along Deep-Time Diversity Oscillation in an Iconic Group of Predatory Insects (Neuroptera: Mantispoidea), Systematic Biology, Volume 74, Issue 3, May 2025, Pages 395–420, https://doi.org/10.1093/sysbio/syae068

**Reviewer:** Here we can see an inconsistency that the limited data provides; *Triatoma rubrofasciata* could not be *Triatoma*, according to your inference.

**A:** Considering that the objective of the analysis was exclusively to investigate the phylogenetic relationships between the species of the *Triatoma infestans* complex and its respective subcomplexes, it was not intended to evaluate whether *Triatoma rubrofasciata* belongs to the genus *Triatoma* or not. A proper definition of the taxonomic position of *T. rubrofasciata* would require a more comprehensive phylogenetic analysis, including all species of the genus, with the incorporation of taxa from North America, the Old World and *Linshcosteus*, to which this species is most closely related (according to Schofield & Galvão, 2009; Justi et al., 2014, 2016; Kieran et al., 2021), in addition to species from the Andes, other regions of South America, and the Spinolai complex.

Reference:

Schofield CJ, Galvão C. Classification, evolution, and species groups within the Triatominae. Acta Tropica. 2009; 110: 88-100.

Justi SA, Russo CA, Mallet JR, Obara MT, Galvão C. Molecular phylogeny of Triatomini (Hemiptera: Reduviidae: Triatominae). Parasites & Vectors. 2014; 7: 137-149.

Justi SA, Galvão C, Schrago CG. Geological changes of the Americas and their influence on the diversification of the Neotropical kissing bugs (Hemiptera: Reduviidae: Triatominae). Plos Neglected Tropical Diseases. 2016; 10 (4): 1-22.

Kieran TJ, Gordon ERL, Zaldívar-Riverón A, Ibara-Cerdeña CN, Glenn TC, Weirauch C. Ultraconserved elements reconstruct the evolution of Chagas disease vectoring kissing bugs (Reduviidae: Triatominae). Systematic Entomology, 2021; 1-16.

**Reviewer:** We can see some inconsistencies here. This needs to be explained in the discussion since it contradicts more recent work.

**A:** The suggestions were included in the text.

# Reviewer report: REVIEWERS COMMENTS

## REVIEWER #1

No comments

## REVIEWER #2

No comments
